# *Lactiplantibacillus plantarum* strains KABP011, KABP012, and KABP013 modulate bile acids and cholesterol metabolism in humans

**DOI:** 10.1093/cvr/cvae061

**Published:** 2024-03-25

**Authors:** Teresa Padro, Victoria Santisteban, Pol Huedo, Montserrat Puntes, Meritxell Aguiló, Jordi Espadaler-Mazo, Lina Badimon

**Affiliations:** Cardiovascular Program-ICCC, Institut d’Investigació Biomèdica Sant Pau (IIB SANT PAU), Sant Antoni Mª Claret 167, Barcelona 08025, Spain; Centro de Investigación Biomédica en Red Cardiovascular (CIBER-CV), Instituto de Salud Carlos III, Av. Monforte de Lemos, 3-5, 28029 Madrid, Spain; Cardiovascular Program-ICCC, Institut d’Investigació Biomèdica Sant Pau (IIB SANT PAU), Sant Antoni Mª Claret 167, Barcelona 08025, Spain; School of Pharmacy and Food Sciences, University of Barcelona (UB), Barcelona, Spain; R&D Department, AB-Biotics S.A. (Part of Kaneka Corporation), Barcelona, Spain; Basic Sciences Department, Universitat Internacional de Catalunya, Barcelona, Spain; Medicament Research Center (CIM), Institut d’Investigació Biomèdica Sant Pau (IIB SANT PAU), Barcelona, Spain; R&D Department, AB-Biotics S.A. (Part of Kaneka Corporation), Barcelona, Spain; R&D Department, AB-Biotics S.A. (Part of Kaneka Corporation), Barcelona, Spain; Cardiovascular Program-ICCC, Institut d’Investigació Biomèdica Sant Pau (IIB SANT PAU), Sant Antoni Mª Claret 167, Barcelona 08025, Spain; Centro de Investigación Biomédica en Red Cardiovascular (CIBER-CV), Instituto de Salud Carlos III, Av. Monforte de Lemos, 3-5, 28029 Madrid, Spain; Cardiovascular Research Chair, Universitat Autònoma de Barcelona, Plaça Cívica, 08193 Bellaterra, Barcelona, Spain

**Keywords:** Small LDL, LDL oxidative capacity, ApoB, Microbiota, Bile acid deconjugation

## Abstract

**Aims:**

Probiotics with high bile salt hydrolase (BSH) activity have shown to promote cardiovascular health. However, their mechanism(s) of action remain poorly understood. Here, we performed a pilot exploratory study to investigate effects of a 4-week intervention with escalating doses of a BSH-active formula containing *Lactiplantibacillus plantarum* strains KABP011, KABP012, and KABP013 on bile acid (BA), lipid profile, and lipoprotein function.

**Methods and results:**

Healthy overweight individuals were included in this study. The probiotic intake was associated with a progressive decrease of conjugated BAs in serum, due to the reduction of tauro- and glyco-conjugated forms. Plasma levels of fibroblast growth factor-19 were significantly reduced and correlated with BA changes. The probiotic induced significant changes in serum lipids, with reduction in non-HDL cholesterol (non-HDLc) and LDL cholesterol (LDLc) levels. The largest decrease was evidenced in the subgroup with higher baseline LDLc levels (LDLc > 130 mg/dL). Fasting levels of circulating apolipoprotein(Apo) B100 and ApoB48 were significantly reduced. Importantly, the decrease in non-HDLc levels was associated with a significant reduction in small LDL particles. Functional testing indicated that LDL particles had a significantly lower susceptibility to oxidation, while HDL particles gained antioxidant capacity after the probiotic intake. The microbiota profile in faeces collected at the end of the study was enriched with members of class *Desulfovibrio*, a taurine-consuming bacteria, likely because of the increase in free taurine in the gut due to the BSH activity of the probiotic.

**Conclusion:**

The intervention with *L. plantarum* strains induces beneficial effects on BA signature and lipoprotein profile. It reduces ApoB and small LDL levels and LDL susceptibility to oxidation and increases HDL antioxidant capacity. These metabolic profile changes suggest increased protection against atherosclerotic disease.

## Graphical Abstract

**Graphical Abstract:**
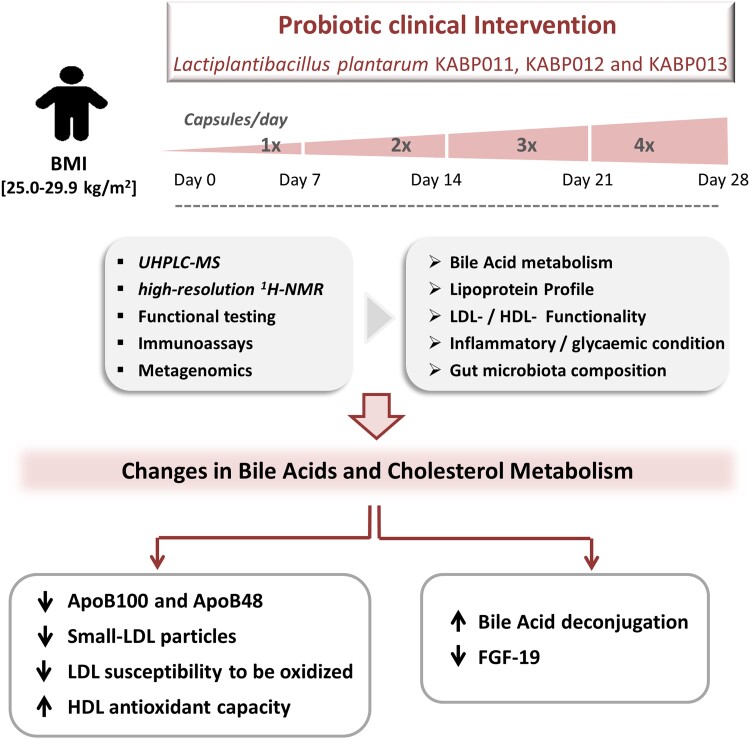

**Time of primary review: 60 days**

## 1. Introduction

Atherosclerotic cardiovascular diseases (ACVD) are the first worldwide cause of death. Among cardiovascular risk factors (CVRF), cholesterol transported by LDL (LDLc) constitutes one of the modifiable factors that have an important impact in the prevention of ACVD progression. In addition to the amount, the LDL particle size is also a key determinant in ACVD progression.^1^ Small and dense LDL particles have an increased capacity to penetrate the arterial wall and are more readily oxidized compared with the large and buoyant particles,^2^ therefore contributing to atherosclerotic plaque progression. Different studies support that individuals with a high amount of small density LDL (sdLDL) are at greater risk of atherosclerotic CVD and myocardial infarction.^3^

Dietary and lifestyle recommendations are often used as a first line of action in the treatment of hypercholesterolaemia.^4,5^ In those individuals who qualify for pharmacological therapy according to existing guidelines, statins are the standard treatment to decrease high plasma LDLc levels and thus the risk of CVD.^6^ However, non-pharmacological therapies are recommended as a first step in the management of hypercholesterolaemia despite a healthy diet and overall lifestyle in individuals.

The potential role of probiotics in the regulation and control of lipid metabolism has raised much interest in the recent years.^7,8^ Probiotics are defined as live microorganisms that when administered in adequate amounts confer a health benefit to the host.^9^ However, different probiotic strains present unique characteristics and consequently strain-specific effects should be considered when searching for specific indications for administration as supplements with therapeutic properties.^10^

The effects of probiotics on cholesterol lowering seem to be mediated by the modulation of the bile acid (BA) metabolism in the gut.^11,12^ However, further understanding of the mechanisms involved in cholesterol lowering and on the effects of strain-specific probiotics is needed to advance in their rightful use in human health protection.

BAs are molecular products derived from the catabolism of cholesterol. They are synthesized in the liver (primary BA) and are typically combined with amino acids glycine or taurine (primary conjugated BA) before secretion into the duodenum to facilitate lipid absorption.^13^ Conjugated BA can behave as signalling molecules regulating systemic endocrine functions including triglyceride, cholesterol, and possibly glucose homeostasis and have direct antimicrobial effects.^11,14^ BAs are efficiently conserved under normal conditions by means of the enterohepatic circulation. Primary BAs can undergo modification by the gut microbiota and generate secondary and tertiary forms through dehydroxylation, dehydrogenation, and sulphation.^11^ Importantly, members of the healthy gut microbiota initiate BA catabolism through an ubiquitous reaction catalysed by bacterial bile salt hydrolase (BSH) enzymes^15^ that ends in the production of unconjugated BAs.

Specific probiotics, in particular *Lactobacillus* species (*sensu lato*), have shown BSH activity.^16^ This activity could influence lipid metabolism and cholesterol levels in the host through the activation of specific signalling pathways.^14,17^ BSH deconjugates bile salts, which are then poorly absorbed, inducing the activation of *de novo* synthesis of bile salts in the liver, leading to higher mobilization of cholesterol stores, and promoting the reduction of plasma cholesterol levels.^17^ This process implies a downregulation of key regulators of energy and lipid metabolism such as the nuclear farnesoid X receptor (FXR)/fibroblast growth factor-19 (FGF-19) feedback loop in humans (FXR/FGF-15 in mice).^17^

*Lactiplantibacillus plantarum* (*L. plantarum*) strains KABP011, KABP012, and KABP013 conform the probiotic formula AB-LIFE® that has shown to reduce total cholesterol (TC) and LDLc in experimental animal studies.^18^ A preliminary study with this probiotic in adults with secondary hypercholesterolaemia showed reduction on TC levels after a 12-week intervention.^19^ The combination of these *L. plantarum* strains (KABP011, KABP012, and KABP013) has previously shown high BSH activity compared with other reference strains and a high capacity to remove cholesterol from media in *in vitro* studies in the presence of bile salts.^20^ These findings lead to the hypothesis that AB-LIFE® effects on cholesterol lowering may be induced by changes in the BA profile produced by the regular oral consumption of the probiotic.

Based on this evidence, we designed a mechanistic study to investigate whether 4 weeks of intervention with the *L. plantarum* formulation containing the strains KABP011, KABP012, and KABP013 (AB-LIFE®) would modulate the BA pool and regulate systemic parameters as circulating lipoproteins and inflammatory molecules when administered to healthy but overweighed volunteers.

## 2. Methods

For more detailed information, see Supplementary material online, Materials and Methods.

### 2.1 Subjects

Healthy volunteers with overweight [body mass index (BMI) 25.0–29.9 kg/m^2^] between the ages of 25 and 60 years (*N* = 20, 10 men and 10 women) were included in the study. Subjects with chronic diseases, treated with lipid-lowering drugs, beta-blockers, diuretics, antibiotics, or with a history of CVD, were excluded (see online supplementary material for more details).

### 2.2 Study design

The intervention trial consisted of a single-centre, single-arm, dose-escalation longitudinal study with 4-week intervention period after a 2-week run-in period (see Supplementary material online, *Figure S1*). Thus, all subjects underwent four sequences of 7 days of treatment with increasing dose of a combination of three *L. plantarum* strains KABP011 (CECT 7527), KABP012 (CECT 7528), and KABP013 (CECT 7529) that refers to a commercially available product (AB-LIFE®).

Each capsule contains 1.2 × 10^9^ colony-forming units (CFU). The lowest dose of probiotic mix, consisting of one capsule per day for the first week, was selected on basis to a previous randomized clinical trial study in hypercholesterolaemic subjects that showed the efficacy of this dose of *L. plantarum* KABP011, KABP012, and KABP013 after 6 and 12 weeks of administration in reducing plasma cholesterol levels.^19^ From the first week on, doses were escalated weekly at the rate of ×2, ×3, and ×4 of the initial dose in order to investigate whether a dose-dependent effect could be identified.

The study was approved by the ethics committee of Hospital Santa Creu i Sant Pau (code IIBSP-PRO-2019-122) and was registered in Clinicaltrials.gov (NCT05378230). Informed written consent was obtained from all participants before their inclusion in the study.

### 2.3 Biological samples

Fasting blood samples were collected at baseline (Day 0) and at the end of each intervention period (Days 7, 14, 21, and 28) from 8 to 11 h a.m., both without anticoagulant and with citrate- and ethylenediamine tetraacetic acid (EDTA)-containing vacutainer tubes. A spot stool sample for each individual was directly collected in OMNIgene-GUT tube (microbiota analysis), as described by the provider, within the 24 h prior to Day 0 (Day −1) and within the 24 h prior to Day 28 (last day of the study).

### 2.4 Anthropometric data, blood pressure, serum lipid profile, and other biochemical measurements

Anthropometric measurements, blood pressure, serum lipid profile, and biochemical measurements were determined at baseline and at Days 7, 14, 21, and 28 (see Supplementary material online, *Figure S1*). Serum biochemical measurements were performed at the centralized laboratory for analysis of the Hospital de Sant Pau using routine commercially available assays for glucose, total BAs, hepatic and renal markers, C-reactive protein (CRP), and standard serum lipid profile (triglycerides, TC, and HDLc). LDL and very LDL (VLDL) cholesterol were calculated using the Friedewald equation. Thyroid hormones were determined by chemiluminescent immunoassays and lipoprotein A [Lp(a)] by immunoturbidimetry.

### 2.5 Analysis of BA profile in serum and faeces

BA profile (primary/secondary and unconjugated/conjugated BA) was analysed in serum and faecal samples by ultra-high performance liquid chromatography–mass spectrometry (UHPLC-MS) after metabolite extraction in methanol^21^ and data processed using the TargetLynx application manager (Waters Corp.),^22^ summarized in Supplementary material online, *Figure S2*.

### 2.6 Metabolic profile of lipoproteins, glycoproteins, lipids, and low molecular weight metabolites by ^1^H-nuclear magnetic resonance

Number and size of circulating lipoproteins of very low-, low-, and high-density (VLDL, LDL and HDL), glycoproteins (Glyc), low molecular weight metabolites (LMWM), and short-chain fatty acids (SCFA) were measured by high-resolution ^1^H- nuclear magnetic resonance (NMR) spectroscopy, using the Liposcale® test (Biosfer Teslab),^23^ with a longitudinal eddy-current delay (LED) pulse spectra as previously described by Mallol *et al.*^24^

### 2.7 Lipoprotein functionality

Purified fractions of LDL (density range 1.019–1.063 g/mL) and HDL (density range 1.063–1.210 g/mL) were obtained from plasma EDTA from individual samples at baseline and at Days 14 and 28 during the probiotic intervention by sequential ultracentrifugation, as previously described.^25^ LDL to be used in the total radical trapping potential (TRAP) assay were isolated from a pool of plasma obtained from normolipaemic subjects and obtained as described above.

LDL susceptibility to oxidation was assessed in plasma-purified LDL by *in vitro* incubation with cupric ions and expressed as the maximal amount of generated conjugated dienes (CDmax) and the time to achieve the half-maximum oxidation value (time to half-maximum).^25^ The antioxidant potential of HDL by performing the TRAP test, which consisted in measuring the change in susceptibility of LDL to oxidation (% of oxidation of LDL particles) in the presence of HDL, as previously described.^25,26^

The HDLc efflux capacity was determined *in vitro* in cholesterol-loaded murine macrophages, as previously described.^26,27^

### 2.8 Immunoassays

Commercial sandwich-based enzyme-linked immunosorbent assay (ELISA) kits were used to measure serum levels of apolipoprotein B-48 (ApoB48) and B-100 (ApoB100) (Cloud-Clone Corp.), adiponectin, insulin, and interleukins (IL) IL-1β and IL-6 (R&D Systems), leptin, and FGF-19 (ABCAM).

Plasma level of the ILs IL-8, IL-12, IL-17A, and tumour necrosis factor-alpha (TNF-α) were quantified using the Millipore’s MILLIPLEX MAP high-sensitivity human cytokine kit (Millipore Corporation, Billerica). All procedures were performed according to the manufacturer’s instructions.

### 2.9 Trimethylamine N-oxide

Trimethylamine N-oxide (TMAO) analysis was performed by UHPLC coupled to a triple–quadruple mass spectrometer with an electrospray ion source (LC-ESI-QqQ) working in positive mode. EDTA plasma samples were extracted in acetonitrile/methanol before analysis.

### 2.10 Microbiota analysis

DNA was extracted from faecal samples at Days 0 and 28 with the MoBio’s Soil DNA Isolation kit (Qiagen). Bacterial 16S rRNA genes were amplified in the V3–V4 region (515F and 806R) and sequenced with MiSeq (Illumina). Fastq files were quality-filtered and clustered into operational taxonomic unit (OTUs) using the QIIME2 software package and classified using a Bayesian classifier trained on the Silva database v.138. Several diversity metrics were computed: number of OTUs and Shannon index for alpha diversity and Bray–Curtis and Jaccard indexes for beta diversity. Changes in alpha diversity between Days 0 and 28 were assessed with Wilcoxon test for paired samples, while changes in beta diversity were assessed by principal coordinate analysis (PCoA) and PERMANOVA. Finally, differential abundance of taxa was assessed by Wilcoxon test for paired samples, using a false discovery rate (FDR) threshold of 0.1^28^ to correct for multiplicity of analysis at each taxonomic level.

### 2.11 Characterization of BSH activity of AB-LIFE strains

BSH activity of the *L. plantarum* formula (strains KABP011, KABP012, and KABP013; 1:1:1) was measured *in vitro*. Shortly, glyco- and tauro-conjugated BA (5 mM) including glycocholic acid (GCA), taurocholic acid (TCA), glycochenodeoxycholic acid (GCDCA), and taurochenodeoxycholic acid (TCDCA) were incubated in the presence of 1 × 10^8^ CFU bacterial suspension (*L. plantarum* strains) in MRS medium 0.5× and anaerobiosis for 90 min. BSH activity was determined by measuring levels of free taurine and glycin at the end of the incubation period using commercial kits (Abcam), following manufacturer’s instructions. Negative control suspensions included MRS 0.5×, MRS 0.5× + BS (5 mM), and MRS 0.5× + bacteria (1 × 10^8^ CFU), and their absorbance values were subtracted from tested suspensions. Control positive suspensions contained MRS 0.5× + taurine or glycine (5 mM), and their absorbance values were considered as 100% deconjugation activity.

### 2.12 Statistical analysis

Data are expressed as median and interquartile range (IQR) for the quantitative variable. Individual average changes were calculated as the mean of the changes for each variable and subject at the end of each intervention period compared with baseline.

Statistical analysis for differences was performed by non-parametrical tests for independent groups, pair comparisons, multiple repeated measurements, or parametric one-sample *t*-test. Correlations between continuous variables were assessed by the Spearman or Pearson coefficients, as indicated. Statistical analyses were conducted using STATA 15 and StatView 5.0.1 software. *P*-values (two-sides) < 0.05 were considered significant.

## 3. Results

### 3.1 Clinical and biochemical characteristics at baseline and after probiotic intervention

Twenty subjects recruited for the study (10 men and 10 women) with median age (Q1; Q3) of 44 (37.5; 47.5) years completed the 4-week intervention period and were included in the analysis. Supplementary material online, *Table S1*, shows the characteristics of the study population (anthropometric, haemodynamic, hepatic, and renal variables) at baseline, after the run-in period (Day 0), as well as at the end of each phase of the study during the 4 weeks of intervention (Days 7, 14, 21, and 28).

Subjects included in the study were overweight, with a median value of BMI of 26.5 (25.4–29.7) kg/m^2^ and a median waist circumference of 97.5 (86.0; 106.0) cm in men and 89.5 (78.0; 92.0) cm in women. None of the participants had chronic diseases and/or cardiovascular risk factors, nor were they under pharmacological treatment (causes of exclusion).

Consumption of probiotic product was well-tolerated, and none of the participants declared major health issues during the intervention period. According to the participant’s response to the weekly telephone call controls and the returned personal diary and capsule containers, compliance was judged as >99% in each intervention period.

Effects of the probiotic intervention on anthropometric parameters did not appear to have clinical relevance. BMI was maintained stable within the overweight range (25.0–29.9 kg/m^2^) during the intervention period, whereas the waist circumference showed a significant decreasing trend (*P* = 0.025).

Haemodynamic parameters such as systolic and diastolic blood pressure (mmHg) and cardiac frequency (beats/min) remained within the normal physiological range during the intervention period. In addition, 4-week intervention with the *Lactiplantibacillus* spp. mixture did not induce major changes on biochemical markers of liver [alanine aminotransferase (ALT), aspartate aminotransferase (AST), GGT], kidney (urea, urate), and muscle (creatine kinase) function that remained within the normal physiological range. Modest changes, within the normal range, were observed in glucose and creatinine levels during the intervention period (*P* < 0.05). However, these did not consistently associate with the dose of probiotic or length/dose of the intervention period.

### 3.2 Effects of the probiotic intervention on BAs

**Figure 1:**
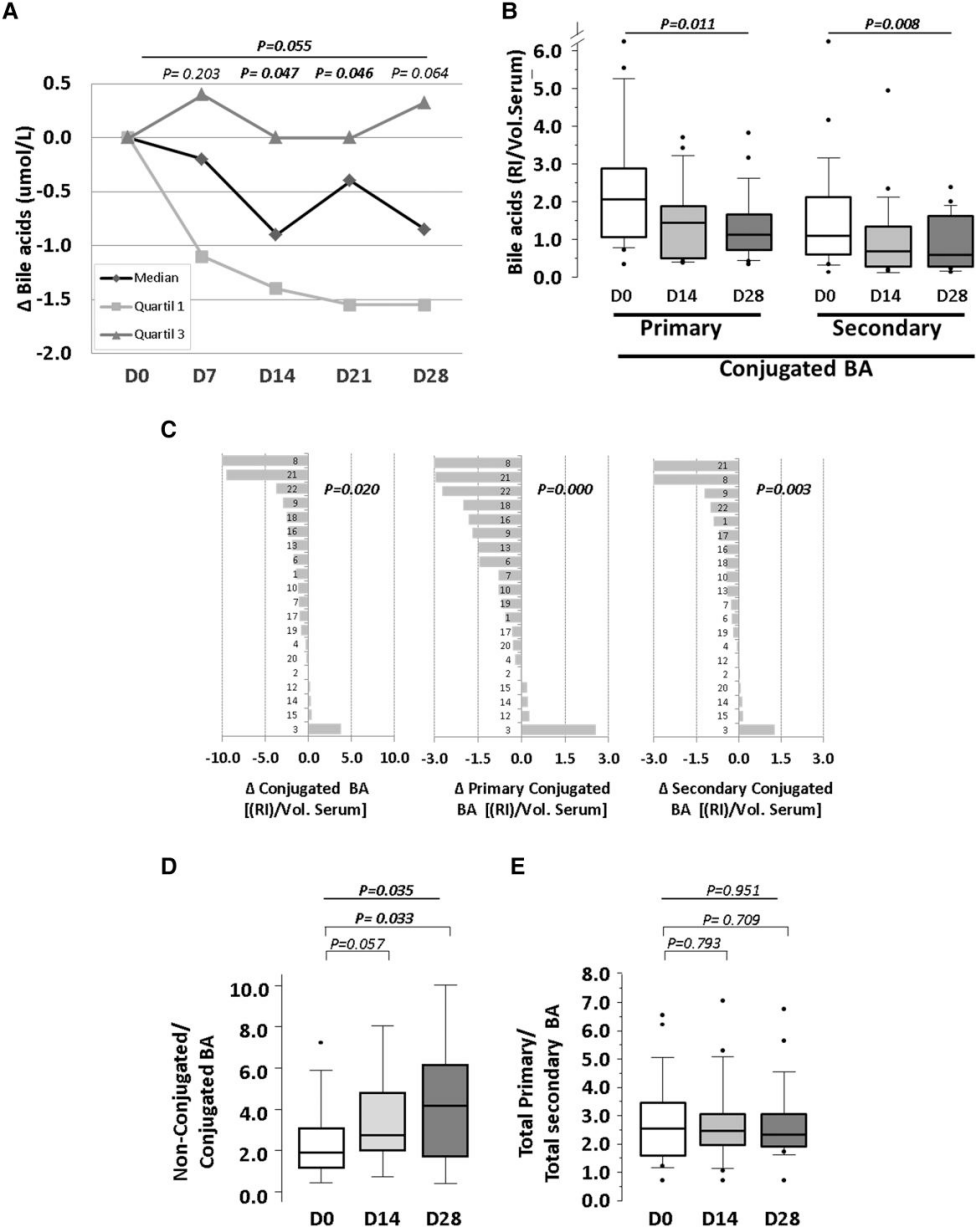
Effect of the 4-week probiotic intervention on serum BA levels. (*A*) Cell line chart of median, Quartile 1, and Quartile 3 of total BAs measured by colorimetric enzymatic assays. (*B*) Box plot of conjugated BAs measured by UHPLC-MS. (*C*) Individual average change of total conjugated BA and primary and secondary conjugated BA. (*D*) Box plot of the ratio of non-conjugated/conjugated BA and (*E*) the ratio of total primary/total secondary BA. *P*-value: (*A*) Wilcoxon signed-rank test. (*B*) Friedman non-parametric test for repeated measures. (*C*) Student’s *t*-test for one sample. (*D* and *E*) Wilcoxon signed-rank test and Friedman non-parametric test for repeated measures. Statistical significance: *P < 0.05*. *N* = 20 for all time points in each panel. D, day; RI, relative intensity; Vol. Serum, volume of serum; Δ, change.

Total levels of BAs in serum were determined by colorimetric enzymatic assays at the end of each intervention period (see Supplementary material online, *Figure S1*). When individual longitudinal changes in BA contents were analysed, a consistent decrease was observed from Day 7 of the intervention achieving statistical significance after the second week of probiotic intake (*Figure 1A*).

UHPLC-MS analysis of BA in serum led to the detection of unconjugated and taurine- and glycine-conjugated species (see Supplementary material online, *Table S2*). Serum levels of non-conjugated BAs did not change, while conjugated BAs were significantly reduced in response to the probiotic intake (*Figure 1B* and Supplementary material online, *Table S2*), with a progressive decrease during the intervention period (Day 14, 1.4-fold decrease vs. baseline, *P* = 0.007; Day 28, 1.7-fold decrease vs. baseline, *P* = 0.006). Among the conjugated BA, primary and secondary forms showed similar response to the probiotic intervention (*P* = 0.011 and *P* = 0.008, respectively), with more than 75% of the subjects showing decreased median values for both BA subtypes (*Figure 1C*).

The ratio between non-conjugated BA and conjugated BA was increased 1.5-fold (*P* = 0.057) and 2-fold (*P* = 0.033) at Days 14 and, 28 respectively (*Figure 1D*), whereas no significant changes were evidenced when the ratio between primary and secondary BA was calculated (*Figure 1E*). Circulating levels of primary BA significantly correlated with those of secondary BA (Rho: 0.875, *P* < 0.001); similar correlations were evidenced when non-conjugated and conjugated BA were analysed separately (Rho: 0.800 and 0.750, respectively, *P* < 0.001).

Excretion of BA in the faeces was comparatively analysed at baseline (Day 0) and Day 28, at the end of the intervention period. The amount of non-conjugated BA in faeces (relative intensity/mg dry tissue) was significantly higher than the amount of conjugated BA (*P* = 0.048), with a similar ratio at baseline and at the end of the intervention period (2.3-fold and 2.2-fold, respectively; Supplementary material online, *Table S3*). The non-conjugated secondary BA was the most abundant fraction in the faeces, with 2- to 3-fold higher amount compared with the primary BA. Lithocholic and 12-oxilithocholic acids, undetected in serum, represented 32.7% and 33.1% (median values), respectively, of the non-conjugated secondary BA in faeces. In contrast, deoxycholic acid, the most abundant secondary BA in serum, only represented 3.7% of the unconjugated secondary BA in the faeces.

The amount of the BAs per mg faeces did not change significantly after the probiotic intervention, being the 12-oxilithocholic acid the only BA metabolite with a decreasing trend at Day 28 (*P* = 0.052 vs. baseline).

BSH activity of the probiotic formula against the four major human BA was analysed in *in vitro* (see Supplementary material online, *Figure S3*). Probiotic mixture displayed a deconjugation activity of 22%, 20%, 2%, and 3% for GCA, GCDCA, TCA, and TCDCA, respectively.

### 3.3 Effects of the probiotic intervention on FGF-19 plasma levels

**Figure 2:**
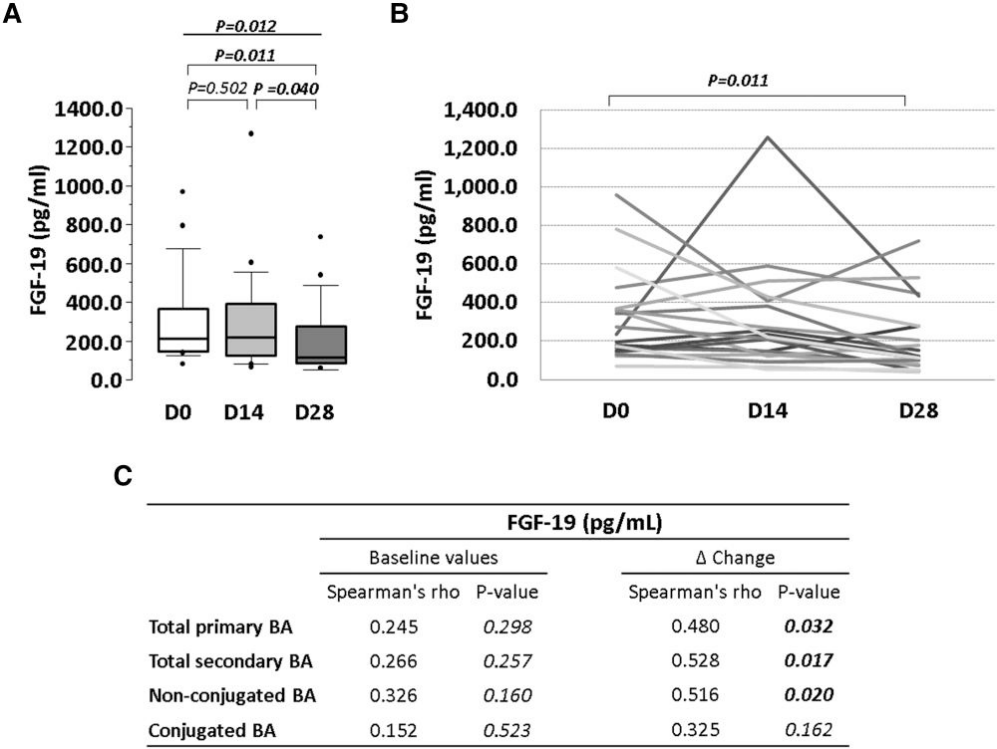
Effect of the 4-week probiotic intervention on FGF-19 levels in plasma. (*A*) Box plot of FGF-19 levels during intervention period. (*B*) Cell line chart of FGF-19 levels of each individual. (*C*) Table of spearman correlation of FGF-19 average change with average change of total primary, total secondary, non-conjugated, and conjugated BA. *P*-value: (*A*) Friedman non-parametric test for repeated measures, (*A* and *B*) Wilcoxon signed-rank test, and (*C*) Spearman’s rank correlation coefficient tests. Statistical significance: *P < 0.05*. *N* = 20 for all time points in each panel. FGF, fibroblast growth factor; BA, bile acids; D, day.

Quantitative analysis (ELISA assay) of plasma FGF-19 at the systemic level showed a decreasing trend during the probiotic intervention, being the levels of FGF-19 at the end of the intervention period significantly reduced compared with levels at baseline (*P* = 0.011) and at Day 14 (*P* = 0.040) (*Figure 2A* and *B*). Plasma baseline FGF-19 values did not correlate with BA, whereas significant correlations were found between changes in BA and FGF-19 in response to the probiotic intervention (*Figure 2C*).

### 3.4 Effects of the 4-week probiotic intervention on serum lipid profile

The median serum lipid concentrations are summarized in Supplementary material online, *Table S4*, for baseline and each intervention period (Days 7, 14, 21, and 28).

**Figure 3:**
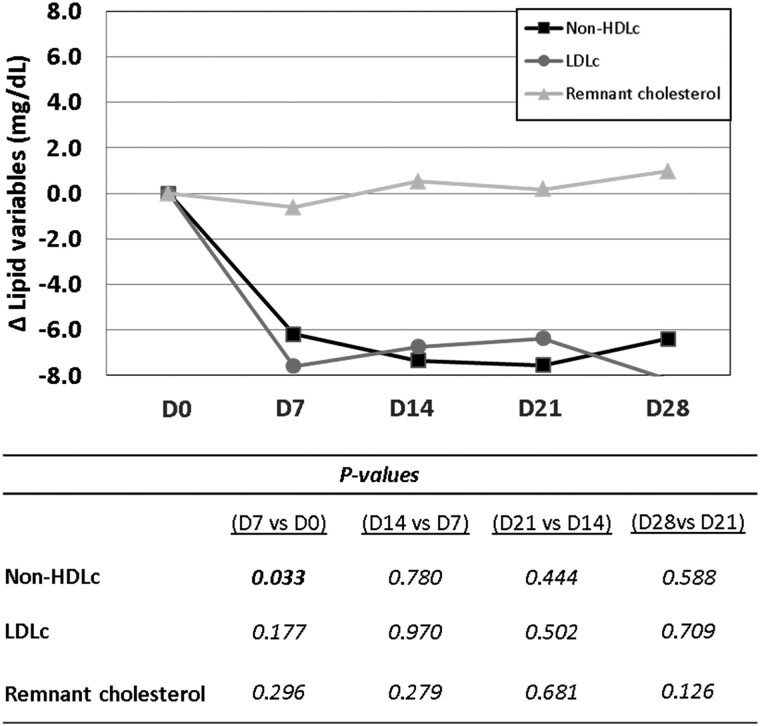
Effect of the 4-week probiotic intervention on lipid levels. Cell line chart representing the median change with respect to baseline of serum non-HDLc, LDLc, and remnant cholesterol during probiotic intervention period. Table *P*-values correspond to Wilcoxon signed-rank test between change at one point with respect to baseline vs. change at prior point with respect to baseline, i.e. (D14–D0) levels vs. (D7–D0) levels. Statistical significance: *P < 0.05*. *N* = 20 for all time points. Δ, change; D, day; HDLc, high-density lipoprotein cholesterol; LDLc, low-density lipoprotein cholesterol.

Individual changes in non-HDLc and LDLc levels showed a decreasing trend during the intervention period. Compared with baseline, the largest decrease in lipid values was found after 1 week intervention, when one capsule of probiotic per day was administered (*Figure 3*), reaching a significant change for non-HDLc [−6.2 (−13.9; 2.9; *P* = 0.033) mg/dL].

**Figure 4:**
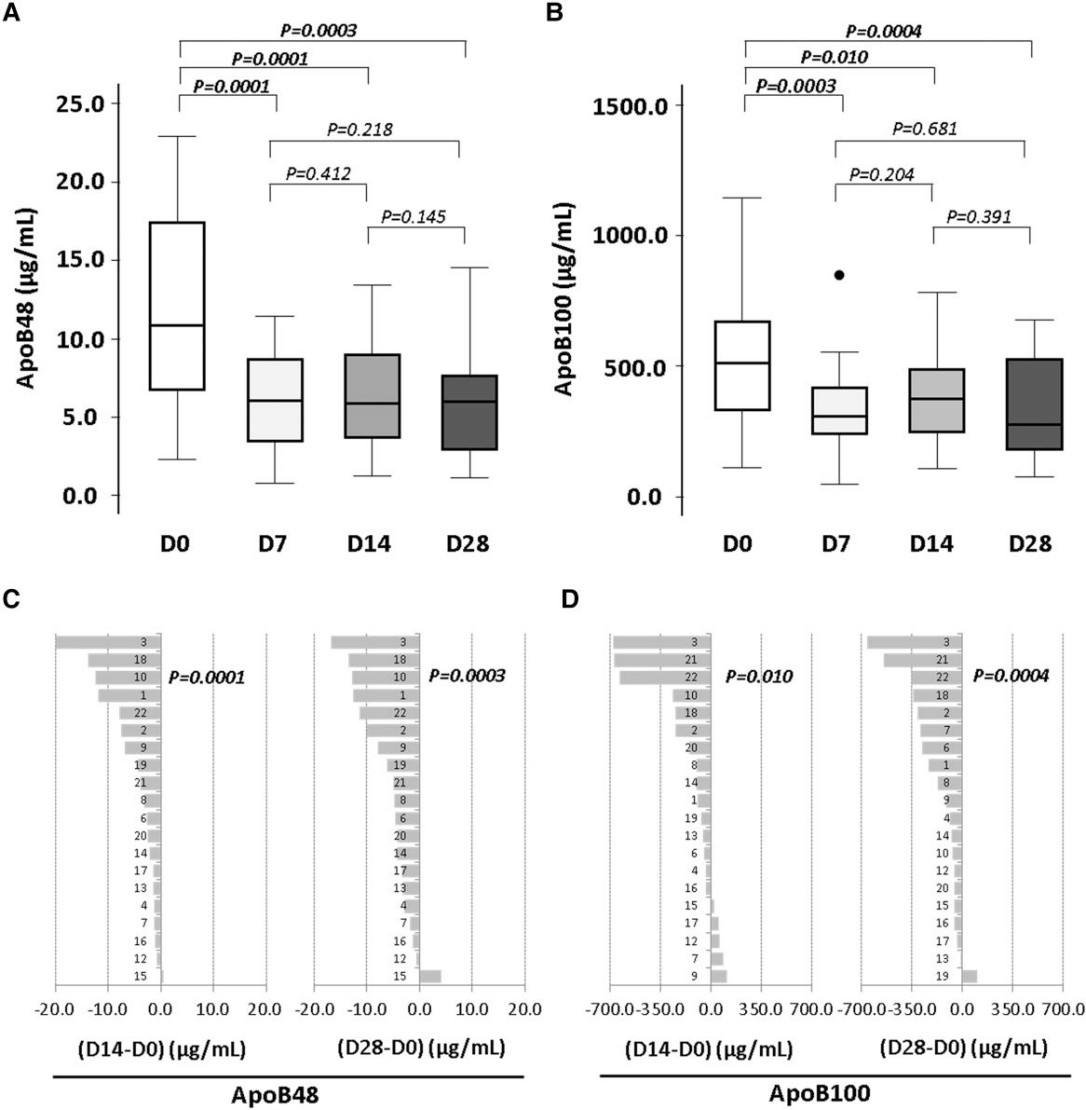
Effect of 4-week intervention with probiotic on ApoB levels. Box plots representing (*A*) ApoB48 and (*B*) ApoB100 levels. Individual average change of (*C*) ApoB48 and (*D*) ApoB100 during probiotic intervention. *P*-value: Wilcoxon signed-rank test. Statistical significance: *P < 0.05*. *N* = 20 for all time points in each panel. Apo, apolipoprotein; D, day.

Fasting plasma ApoB100 and ApoB48 levels were significantly decreased after the first week intervention with the probiotic, and levels were maintained reduced, independently of the administrated probiotic dose throughout the intervention period (*Figure 4A* and *B*). The probiotic-related decrease in ApoB48 was evidenced in 19 of 20 subjects at Days 14 and 28 of the intervention period (*Figure 4C* and *D*).

The study population had median (Q1–Q3) Lp(a) levels of 15.2 (8.1–37.9) mg/dL. No significant changes in the levels of Lp(a) were evidenced during 4 weeks of intervention with the probiotic (see Supplementary material online, *Table S4*).

### 3.5 Differences in the response to the probiotic in relation to the background LDLc level

We tested the effect of the probiotic in the subgroup of patients with higher levels of LDLc at baseline. BMI and lipid profile at baseline in the low- and high-LDL groups are shown in Supplementary material online, *Table S5A*. The high-LDLc group [median 144.6 (139.6; 152.4) mg/dL] showed a larger decrease of non-HDLc and LDLc levels than the low-LDLc group [median 96.5 (80.9; 119.4) mg/dL] after probiotic uptake. The effect was already evident at Day 7 and maintained stable during all the intervention period (see Supplementary material online, *Figure S4*).

No differences were observed for BA subtypes and FGF-19 at baseline between subjects with low and high background LDLc levels (see Supplementary material online, *Table S5B*). Total conjugated BA and their primary and secondary subtypes had a decreasing trend both in the low- and high-LDLc groups but only in the latter achieved statistical significance (see Supplementary material online, *Table S6A*). Changes in FGF-19, although with reducing trend, depicted higher variability when analysed separately in the two LDLc subgroups, differences being statistically significant for subjects with LDLc below 130 mg/dL (*P* = 0.045; Supplementary material online, *Table S6B* and *C*).

### 3.6 Effects of the probiotic intervention on the composition, diameter, and quantity of serum lipoproteins

**Figure 5:**
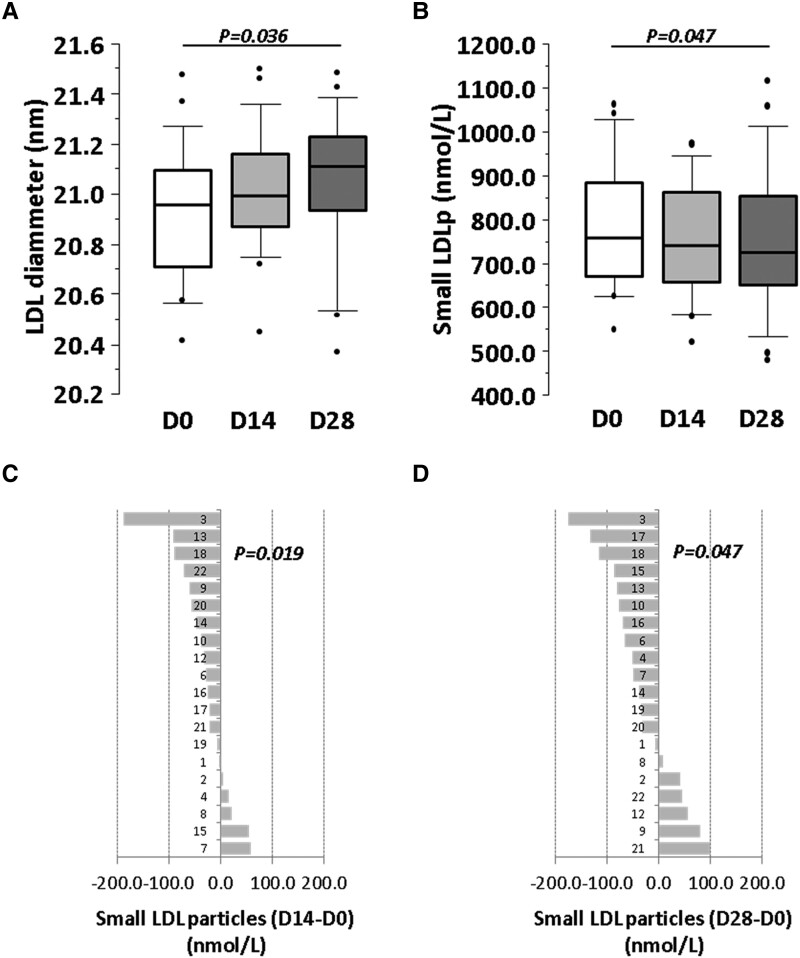
Effect of 4-week intervention with probiotic on LDL lipoproteins. Box plots representing (*A*) LDL diameter and (*B*) small LDL particles concentration at baseline and Days 14 and 28. (*C* and *D*) Individual difference of number of small LDL particles at (*B*) Day 14 and at (*C*) Day 28 with respect to Day 0. *P*-value: (*A* and *B*) Friedman non-parametric test for repeated measures. (*C* and *D*) Student’s *t*-test for one sample. Statistical significance: *P < 0.05*. *N* = 20 for all time points in each panel. LDL, low-density lipoproteins; D, day.

Lipoprotein size (diameter in nm) and quantity (nmol/L) were determined by NMR, at baseline and after 2 and 4 weeks of intervention (see Supplementary material online, *Table S7A* and *B*). The median diameter of the LDL particles was significantly larger at the end of the intervention period (*P* = 0.036) (*Figure 5A*), whereas the diameter of HDL and VLDL particles was not significantly modified during the intervention.

When lipoproteins were sub-grouped according to their size (small, medium, and large), a significant reduction in the number of LDL of small size (diameter: 17.5–20.5 nm) was observed after the 4-week intervention period (*Figure 5B* and Supplementary material online, *Table S7B*). This reduction was evident in >70% of the study population at Day 14 of intervention (*P* = 0.019) (*Figure 5C* and *D*). No changes were detected in the medium- and large-size subgroups of LDL (see Supplementary material online, *Table S7B*), neither in VLDL nor in HDL.

### 3.7 Effects of the probiotic intervention on lipoprotein functionality

**Figure 6:**
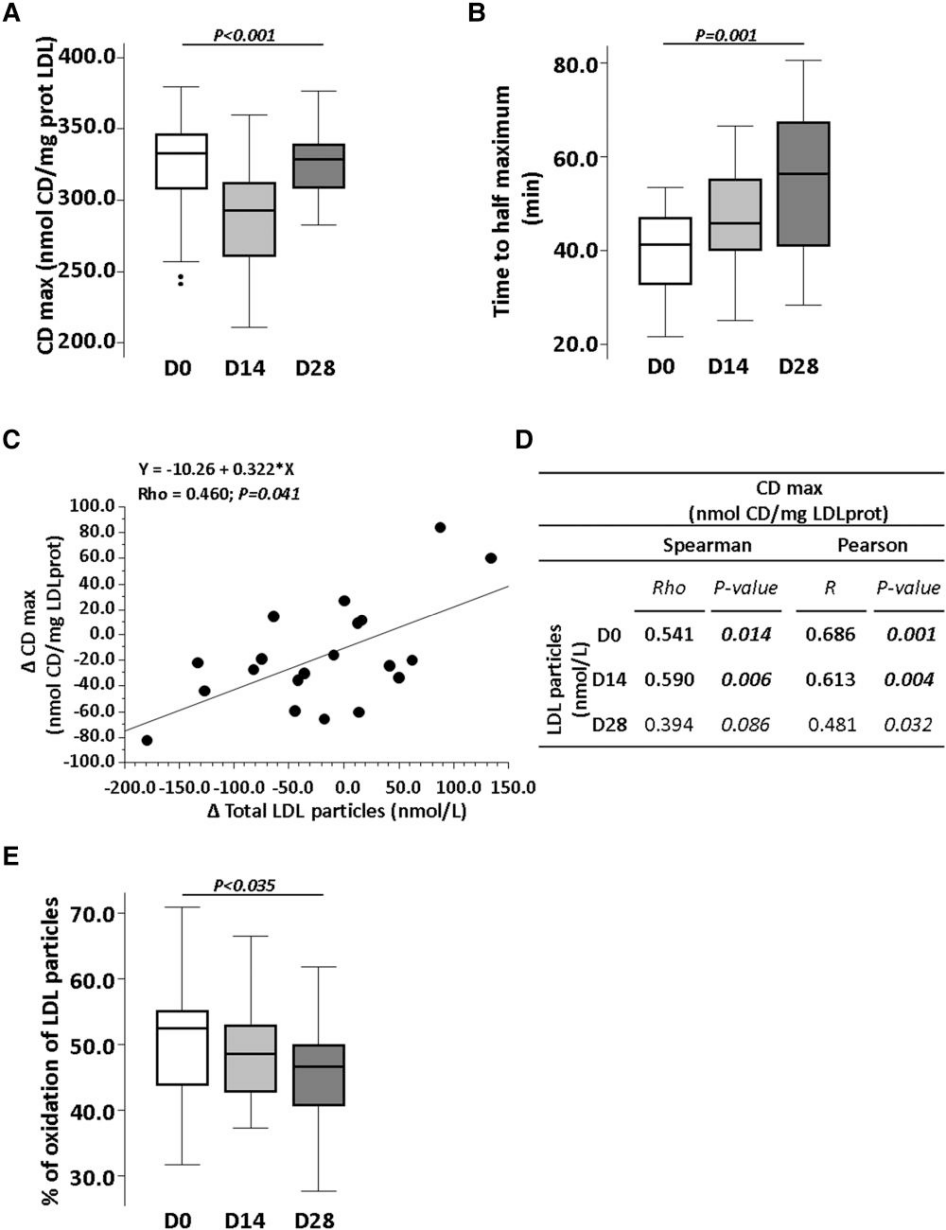
Effect of 4-week intervention with probiotic on lipoprotein functionality. Box plots representing (*A*) conjugated dienes (CDmax) and (*B*) the time to achieve the half-maximum oxidation value (time to half-maximum) at baseline and Days 14 and 28. (*C*) Correlation between conjugated dienes average change and LDL lipoprotein average change represented in bivariate scattergram and Spearman and Pearson coefficients. (*D*) Table with Pearson and Spearman correlation coefficient between conjugated dienes and LDL lipoprotein levels at baseline and Days 14 and 28. (*E*) Box plots representing % oxidation of LDL particles. *P*-value: (*A* and *D*) Friedman non-parametric test for repeated measures and Wilcoxon signed-rank test. (*B* and *C*) Spearman’s rank and Pearson correlation coefficient tests. Statistical significance: *P < 0.05*. *N* = 20 for all time points in each panel. LDL, low-density lipoproteins; D, day; Δ, change.

LDL susceptibility to oxidation was found significantly decreased by the probiotic intervention (*P* < 0.001) (*Figure 6A*). Importantly, in 85% of the participants, *L. plantarum* mixture intake induced a progressive lengthening of the required time to reach the half-maximum LDL oxidation (D14, *P* = 0.019, and D28, *P* < 0.001, both vs. baseline) (*P* < 0.001) (*Figure 6B*).

Correlation analysis (non-parametric Spearman test) revealed that subjects presenting a better response to the probiotic, regarding reduction of LDLc levels, also showed a greater reduction in the susceptibility of LDL to oxidation (Rho = 0.633; *P*-value = 0.003). Moreover, the reduction in LDL susceptibility to oxidation (CDmax) significantly correlated with the decrease in the number of LDL particles (nmol/L) (*Figure 6C* and *D*). Thus, the number of LDL particles correlated with CDmax at baseline (Rho: 0.541, *P* = 0.014) and at Day 14 of probiotic intervention (Rho = 0.590, *P* = 0.006). A positive association was also observed at Day 28, although did not achieve significance (Rho: 0.394, *P* = 0.086).

The antioxidant capacity of HDL over the LDL particle oxidation was evaluated at baseline and after 14 and 28 days of intervention. Thus, in the presence of HDL, LDL oxidation at baseline was reduced to 52.5% (43.8; 55.1). This percentage was further reduced to 48.6% (42.8; 53.0) and to 46.7% (40.7; 49.9− (*P* = 0.035) after 2 and 4 weeks of intervention with the probiotic, being the difference at Day 28 statistically significant compared with baseline (*P* = 0.028) (*Figure 6E*).

The capacity of ApoB-depleted serum to induce cholesterol efflux from macrophages *in vitro* was directly correlated to the content in ApoA and number of HDL particles at baseline (% cholesterol efflux vs. ApoA, Rho = 0.599, *P* = 0.005; % cholesterol efflux vs. HDL particles, Rho = 0.496, *P* = 0.026). Four weeks of intervention with probiotic did not significantly modify the percentage of cholesterol efflux mediated by the apoB-depleted serum [D0, 23.5% (20.2; 34.5); D14, 25.1% (20.2; 30.6); D28, 23.2% (20.5; 20.9)].

### 3.8 Effects of the probiotic intervention on glucose metabolism markers and insulin sensitivity index

Insulin, adiponectin, and leptin were quantitatively analysed at baseline and during the probiotic intervention (*Table 1*).

**Table 1:** Changes in glucose metabolism markers and insulin sensitivity index during the 4-week intervention with probiotic

	Baseline	Intervention period		
	Day 0	Day 14	Day 28	*P*-value
**Glucose metabolism markers**				
Insulin (pmol/L)	59.8 (32.8; 89.0)	50.7 (35.7;101.5)	58.7 (32.5; 96.3)	0.157
Leptin (ng/mL)	11.0 (34.2; 15.6)	10.9 (4.9; 15.9)	10.8 (6.8; 15.9)	0.245
Adiponectin (µg/mL)	6.0 (3.8; 9.0)	6.6 (4.0; 8.2)	6.5 (4.8; 9.2)	**0**.**019**
**Insulin sensitivity index**				
HOMA-IR	1.7 (0.9; 2.7)	1.4 (1.0; 3.3)	1.7 (0.9; 2.9)	0.549
HOMA-*β*	135.0 (78.6; 191.2)	139.4 (65.9; 198.8)	175.3 (105.1; 210.5)	**0**.**047**

Values are expressed as median (IQR). *P*-value: Friedman non-parametric test for repeated measures. Statistical significance: *P < 0.05* are shown in bold. *N* = 20 in all time points. HOMA, homeostatic model assessment; IR, insulin resistance.

All variables had values within the normal range at baseline. The probiotic administration induced a significant increase in adiponectin plasma levels at the end of the intervention period (*P* = 0.019). In contrast, no effect of the probiotic intervention was evidenced on plasma insulin and leptin levels.

Two standard homeostatic model assessment indexes were calculated to determine insulin resistance (HOMA-IR), and the pancreatic beta cell function (HOMA-β) and levels at Days 14 and 28 of probiotic intervention were compared with those at baseline (see *Table 1*). The study population had a median value of 1.7 (0.9–2.7) for HOMA-IR and 135.0 (78.6–191.2) for HOMA-β at baseline. Values for HOMA-β were significantly increased at the end of the probiotic intervention [175.3 (105.1–210.5), *P* = 0.047 vs. baseline], suggesting an improvement of the beta cell function.

### 3.9 Effects of the probiotic intervention on inflammatory markers

Plasma levels of CRP at baseline and during the probiotic intervention and those for cytokines IL-1β, IL-6, IL-8, IL-12, IL-17, and TNF-α and Glyc GlycA and GlycB are shown in *Table 2*. The study population depicted a low inflammatory profile, which was not significantly affected by the 4 weeks of intervention period with the probiotic. Plasma levels of the inflammation-related markers were unrelated to the BMI and age in this study population.

**Table 2:** Plasma levels of inflammatory markers during the 4-week intervention with probiotic

	Baseline	Intervention period		
	Day 0	Day 14	Day 28	*P*-value
IL1-β (pg/mL)	0.12 (0.09; 0.16)	0.13 (0.10; 0.21)	0.16 (0.11; 0.22)	0.638
IL-6 (pg/mL)	0.48 (0.07; 1.02)	0.22 (0.00; 0.78)	0.32 (0.00; 0.94)	0.106
IL-8 (pg/mL)	0.6 (0.6; 12.3)	0.6 (0.6; 13.7)	0.6 (0.5; 9.9)	0.332
IL-12 (pg/mL)	1.2 (0.8; 2.1)	1.2 (1.0; 2.1)	1.5 (0.9; 1.6)	0.963
IL-17 (pg/mL)	4.1 (3.0; 8.4)	4.8 (4.1; 7.5)	4.8 (3.3; 8.4)	0.951
TNF-alpha (pg/mL)	4.6 (2.6; 6.8)	4.1 (2.3; 6.1)	4.1 (3.2; 5.1)	0.638
CRP (mg/L)	1.2 (0.5; 2.1)	1.8 (1.1; 3.0)	1.2 (0.5; 2.5)	0.259
GlycA (μmol/L)	683.5 (619.7; 813.1)	701.0 (629.5; 802.9)	740.2 (629.6 ; 770.6)	0.951
GlycB (μmol/L)	357.1 (336.1; 369.6)	356.7 (345.7; 373.2)	363.5 (321.6; 370.1)	0.165

Values are expressed as median (IQR). *P*-value: Friedman non-parametric test for repeated measures. Statistical significance: *P < 0.05*. *N* = 20 in all time points.

Subgroup analysis using as cut-off the mean value for each inflammatory biomarkers (see Supplementary material online, *Table S8*) showed that all variables, except IL-1β and IL-8 that had 70% and 50% of the values below detection at baseline, respectively, presented a decreasing trend (statistically significant for IL-6) after 28 days of intervention with *L. plantarum*. This pattern was not found in the subjects with inflammatory levels below the cut-off value. Differences in the response for subgroups below and above the cut-off were significant for IL-6, IL-12, CRP, and GlycA.

### 3.10 Effects of probiotic on thyroid hormones and TMAO levels

Levels of thyroid hormones and TMAO are given in Supplementary material online, *Table S9*.

Only minor effects on serum levels of thyroid hormones were seen during the probiotic intervention; changes in TSH positively correlated with those in T4 [Rho = 0.569 (*P* = 0.009)] and in T3 [Rho = 0.461 (*P* = 0.041)]. No correlation was found between TSH and T4 or TSH and T3 at baseline.

Median TMAO levels were unchanged by the probiotic uptake. At baseline, 18 of 20 subjects had TMAO levels ≤ 4 µM (physiological range < 6 µM) and 2 subjects presented serum values above 15 µM (high range for CVD risk). At Day 28, TMAO levels were reduced 2–4 fold compared with baseline in these two subjects, while TMAO values in the remaining individuals varied within the physiological range [2.3 (1.3–3.4) vs. 3.4 (1.8–4.9] µM].

### 3.11 Effects of the probiotic on SCFA and LMWM in faeces

The profiles of SCFA and LMWM in faeces were analysed by NMR in dry stool samples at baseline and at the end of the intervention period (see Supplementary material online, *Tables S10* and *S11*).

In the healthy population with overweight, three SCFAs, acetate, propionate, and butyrate, were majority in the faeces, representing more than 97% of the total content, whereas only acetate was consistently found in serum. Four weeks of intervention with probiotic did not significantly change the SCFA content in the stool nor the SCFA acetate concentration in serum (see Supplementary material online, *Table S10*).

In addition, a set of 22 LMWM were consistently detected in faeces. These included aromatic and branch amino acids (54%), metabolites of glucose metabolism (14%), pyrimidine and purine metabolites (9%), and microbial metabolites (9%), among others. As shown in Supplementary material online, *Table S11*, more than 85% of the metabolites presented a negative trend for their content in faeces after the probiotic intervention compared with baseline, but changes did not achieve statistical significance, except for glycine and sarcosine (*P* = 0.014 and *P* = 0.025, respectively). When analysed in serum, glycine levels were within the physiological range (200 and 300 µmol/L)^29^ and did not show significant differences between baseline and Day 28 after probiotic intervention [238.9 (218.5; 283.1) vs. 237.2 (218.5; 277.1) µmol/L, *P* > 0.05]. Sarcosine was under detection level (not detected) in serum.

To better understand metabolite changes in faeces after the probiotic intervention, we performed correlation analysis of 30 metabolites (including SCFA). The generated network (see Supplementary material online, *Figure S5*) revealed an association between 23 metabolites, which distributed in 2 clusters, linked by the interaction between the propionate SCFA and the phenylacetate. The biggest cluster, mostly consisting of two classes of metabolites, amino acids, and SCFAs, showed a high degree of interaction among the metabolite species of the same type (based on Pearson correlation coefficients > 0.80 and a *q*-value for correlation < 0.05), as they are the branched AA leucine–isoleucine–tyrosine, the aromatic AA valine–alanine–glycine, and the SCFAs propionate, acetate, and butyrate.

### 3.12 Effects of the probiotic on the gut microbiota composition

Relative abundance of phyla and classes detected in faecal samples at Days 0 and 28 is represented in Supplementary material online, *Figure S6A* and *B*, and specific data and *P*-values provided in Supplementary material online, *Table S12*.

**Figure 7:**
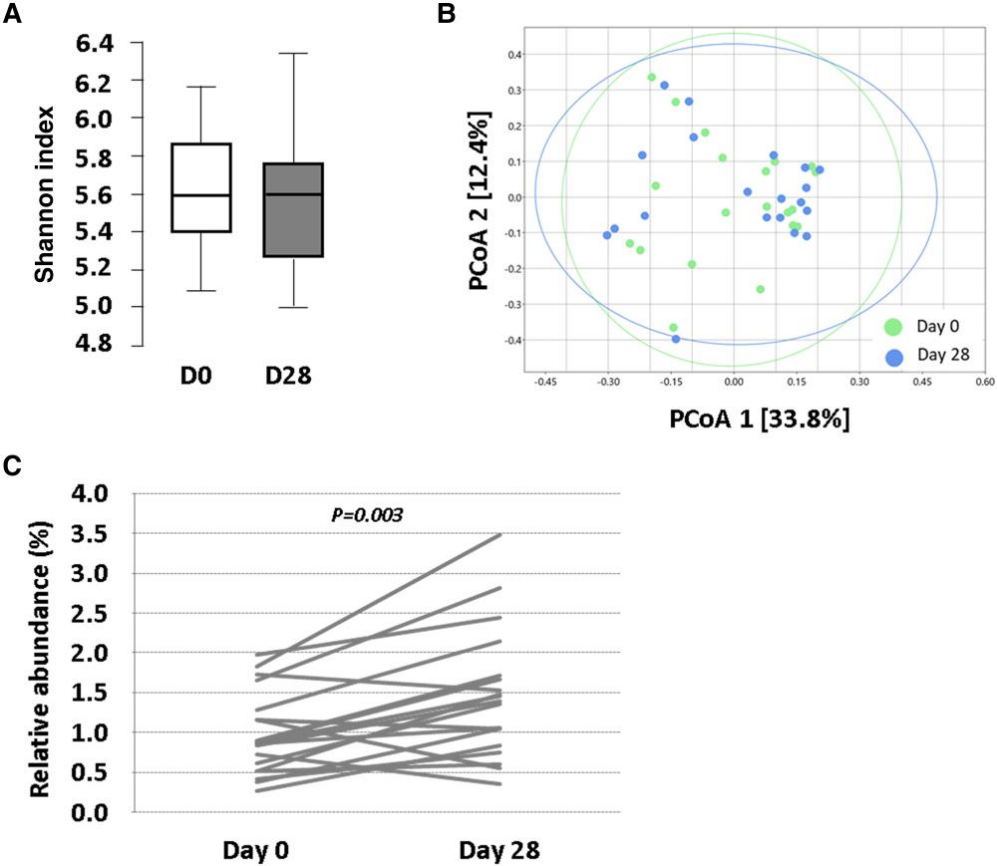
Analysis of microbiota in faecal samples of study participants at baseline and at Day 28. (*A*) Box plot representing of alpha diversity (Shannon index). (*B*) Beta diversity (PCoA based on Bray–Curtis similarity index). (*C*) Cell line chart of relative abundance (%) of class *Desulfovibrio* of each individual. *P*-value: (*C*) Wilcoxon signed-rank test, significance retained upon adjusting for multiplicity at an FDR of 0.10. *P < 0.05*. (*A* and *B*) *N* = 20 for all time points. (*C*) *N* = 20 at Time 0 and *N* = 19 at Time 28 (one subject with levels under detection limit). D, day; PCoA, principal coordinate analysis.

No global changes in alpha and beta diversity were noted. However, analysis of differential abundance in microbiota classes after the probiotic intervention showed a significant increase in the abundance of *Desulfovibrio* (*P* = 0.004; *q* = 0.003, after FDR correction) in faecal samples collected at Day 28, compared with Day 0 (*Figure 7*). This increasing effect was also nominally significant at order and family taxonomical levels (*Desulfovibrionaceae*, *P* = 0.003), although the significance was lost after FDR correction for multiplicity. Besides, nominally significant changes were also noted in orders *Oscillospirales* and unclass_Bacilli_RF39, as well as in the families *Oscillospiraceae* and *Oxalobacteraceae*. In addition, >60% of the subjects in the study depicted an increase of the faecal relative abundance in *Bacteroides* (65%), *Proteobacteria* (65%), and *Actinobacteria* (59%).

Six bacterial phyla (*Bacteroidetes*, *Firmicutes*, *Proteobacteria*, *Verrucomicrobiota*, *Actinobacteria*, and *Desulfobacterota*) represented 97% of the faecal microbial content. All of them, except *Verrucomicrobiota*, were consistently detected in 90–100% of the study population and, therefore, selected to further investigate the association between gut bacterial species and the changes in BAs and ApoB at the end of the probiotic intervention.

As shown in Supplementary material online, *Table S13*, the baseline relative abundance of the five selected phyla was not associated (Spearman correlations) with the changes observed at plasma level in total and conjugated BAs nor in ApoB48 and ApoB100, after 28 days of intervention with *L. plantarum* mixture. However, when individuals were grouped by the response of each bacterial phyla to the probiotic (changes > 0 and <0 with respect to baseline), opposite trend distributions were found for increasing and decreasing response of *Bacteroidetes* and *Actinobacteria* in relation to the tertiles of change in conjugated BA (see Supplementary material online, *Figure S7*).

Decrease in ApoB48 and ApoB100 after the probiotic intervention was unrelated to the response of the gut microbiota (five phyla) to the intervention with the *L. plantarum* mixture.

## 4. Discussion

Conversion of cholesterol to the BAs is a major route for lowering serum cholesterol.^30^ Cholesterol reduction by probiotics seems to be mediated by different mechanisms.^31^ In the case of probiotics with typical BSH activity, the process is likely related to the deconjugation of conjugated BAs.^32,33^ However, the precise mechanisms at play needed to be proved in humans where the relationship between probiotics, BA metabolism, and LDL profile was poorly addressed.

Here, we performed a prospective and interventional study to investigate the effects of a 4-week intervention with escalating doses of a probiotic formulation with high BSH activity comprising *L. plantarum* strains KABP011, KABP012, and KABP013 (1:1:1) on BAs and lipoproteins (composition and function), in healthy subjects with overweight. Our results provide unambiguous evidence that daily intake of this *Lactiplantibacillus* formulation induces a decrease of conjugated BAs, a reduction in ApoB (ApoB100 and ApoB48) levels, a decrease in the small LDL particle number, and an improvement on LDL and HDL particle antioxidative properties. So far, few studies based on clinical interventions with BSH-active probiotics have referred to their effects on the BA signature and on lipid/lipoprotein metabolism, with apparently controversial findings.^34^

The overweight population included in the current study was similar in BMI range to a previous placebo-controlled clinical trial in which the mixture of *L. plantarum* strains resulted in a reduction on total lipid levels after 12 weeks of intervention.^19^ This level of overweight was chosen because it is representative of people with mild hypercholesterolaemia (without the need for pharmacological treatment) that is recommended to follow lifestyle–dietary interventions.^12,35^

*In vitro* experiments showed that *L. plantarum* can deconjugate physiological concentrations of major human taurine- and glycine-conjugated BAs^36,37^ with a preference for the latter. In humans, BA conjugation is catalysed by the BA coenzyme-A ‘BAAT’ (amino acid N-acyltransferase), which seems to have a preference for taurine compared with glycine.^38^ However, human bile contains three times more glycine-conjugated BAs than taurine forms^39^ supporting the notion that taurine availability is a limiting factor for *de novo* BA conjugation. In our study, we observed a higher reduction of tauro-conjugated than glyco-conjugated BA forms.

Free deconjugated BAs are less soluble and therefore less able to be reabsorbed into the enterohepatic circulation and more easily excreted into faeces, enhancing the *de novo* hepatic BA synthesis.^40^ Unfortunately, we only took sample of a spot faecal deposition, which did not allow us to measure the total amount of excreted unconjugated BA before and after the probiotic intervention. Whether the pool of unconjugated BA generated in the small intestine after the probiotic intervention undergoes microbial modifications to undetected forms or they are truly increased in the faeces will require further studies. Likewise, it is interesting to note that faecal secondary BA such as the litocholic acid, mainly produced through bacterial biotransforming reactions in the colon via 7α/7β-dehydroxylation, or the 12-oxo-lithocholic, a BA-derivate produced by intestinal bacteria from a secondary BA, although detected in the faeces, did not change after the intervention period, supporting that the probiotic mixture do not display 7α-dehydroxylation activity^41^ and therefore do not contribute to accumulation of potentially detrimental BA.^42^

FGF-19 is increasingly acknowledged as a key metabolic signalling molecule mediating negative feedback inhibition of BA synthesis. Moreover, the BA/FGF-19 axis has emerged as a relevant endocrine partner in energy metabolism.^43,44^ In this study, we observed a coordinated decrease in FGF-19 and BAs after the probiotic intake suggesting the promotion of a *de novo* synthesis of BA in the liver from circulating cholesterol, due to the reduction of the negative FGF regulatory stimulus. In agreement, experimental data from pigs evidenced a downregulation of ileal and hepatic FGF-19 expression in animals receiving a diet complemented with *Lactobacillus delbrueckii*, which went along with an increase in the expression of cholesterol 7 alpha-hydroxylase (CYP7A1), a rate-limiting enzyme in the BA synthesis pathway^45^

To date, cholesterol-lowering properties of certain probiotics and improvement of blood lipid profiles after several weeks intervention have been especially reported in hypercholesterolaemic conditions.^46^ The specific combination of *L. plantarum* strains used in this study showed to reduce TC and LDLc in hypercholesterolaemic patients after a 12-week intervention.^19^ In our study, a significant reduction of TC and non-HDLc is demonstrated in overweight but otherwise healthy volunteers with no cardiovascular risk factors, right after 1 week of intervention with one capsule/day containing 1.2 × 10^9^ CFUs. Analysis of changes in TC and non-HDLc at the individual level showed a positive response in 75% of the study population, with the largest effect found in the group of subjects with higher cholesterol levels at study initiation (within the normality range as per inclusion criteria).

A novel finding in this study was the reduction in the number of small dense LDL during the probiotic intervention. To our knowledge, this is the first time that a probiotic has proven to have a preferential effect on the reduction of circulating small LDL particles, which is the LDL fraction with higher arterial wall permeability due to their size and therefore the largest pro-atherogenic potential.^1^

ApoB, the primary apolipoprotein in the non-HDL fraction,^47^ was significantly reduced by the probiotic uptake. Both ApoB-100, of hepatic origin, and ApoB-48, expressed in the small intestine,^48^ were significantly reduced just after the first week intervention, and levels were maintained reduced throughout the intervention period, with a similar pattern as the probiotic-induced changes in total BAs. Interestingly, ApoB-100 and ApoB-48 levels significantly correlated with the number of LDL particles and levels of LDLc at baseline and at the different times during the probiotic intervention. These results suggest that the beneficial effects of the probiotic intervention are also mediated by inhibition of the intestinal lipid transport. Further studies are needed to investigate the potential link between the probiotic-induced changes in the BA/FGF-19 axis and the changes in the lipoprotein composition and profile.

Oxidation of LDL is a hallmark of atherosclerosis development. Here, we observed a significantly reduced susceptibility of LDL to become oxidized while promoting the atheroprotective properties of HDL after the probiotic intake. Experimental studies in obese mice models reported that *Lactobacillus* strains reduce circulating levels of the lipid peroxidation reaction product malondialdehyde (MDA),^49,50^ and *in vitro* studies had evidenced that several acid lactic bacteria from *Lactobacillus* and *Streptococcus* genera have the capacity to reduce LDL oxidation.^51^ In addition, 14-week intervention with *Lactobacillus helveticus* reduced Cu^2+^-induced oxidation of LDL particles in elite athletes submitted to strenuous exercise in order to have an increased generation of free radicals and oxidative stress.^52^

Recent clinical studies have reported on the effects of probiotics on several satiety-related factors, including adiponectin and leptin, although with unconclusive findings.^53^ In our study, probiotic administration resulted in a significant increase in adiponectin plasma levels at the end of the intervention period, but it had no effect on plasma leptin levels. Interestingly, adiponectin levels were inversely associated with fasting plasma ApoB48 levels in a cross-sectional study in healthy adolescents,^54^ which is in line with the changes found in these variables in our probiotic intervention study. Adiponectin is secreted by white adipose tissue and seems to have insulin-sensitizing and anti-inflammatory properties.^55^ In our study of volunteers without a pro-inflammatory baseline condition (healthy, non-smoking population with a BMI < 30 kg/m^2^), we did not detect major changes in any of the inflammatory markers investigated, including CRP, TNF-alpha, GlycA, GlycB, and ILs such as IL-1β, IL-6, IL-8, IL-12, and IL-17. Nonetheless, when patients were sub-grouped according to their inflammatory baseline profile for the different variables, those subjects with a more marked inflammatory background were better responders to the *L. plantarum* intervention, with a decreasing trend that achieved statistical significance for the IL-6. In line with our findings, inflammatory markers had shown to be affected by probiotic interventions when investigated in group populations with pro-inflammatory background, as is the case with patients with insulin resistance in type 2 diabetes^56^ or already established stable coronary artery diseases.^57–59^ Interestingly, inflammatory markers were unchanged in a study in normal to mildly hypercholesterolaemic adults who received *L. plantarum* ECGC 13110402 during a 12-week period.^60^

Analyses of faecal microbiota revealed that probiotic intervention had no effect on general microbiota diversity but significantly increased abundance of members of the class *Desulfovibrio*, one of the few gut bacteria able to consume taurine anaerobically,^61^ which could reduce taurine in the BA pool, thus preventing reconjugation of taurine forms, as reported in this study. Interestingly, it has been recently shown that deconjugated BA prevents colonization of the pathogen *Clostridium difficile* in mice, in which dominate taurine forms.^62^ In agreement to our findings, gut microbiota is recognized as a highly resilient ecosystem, reflecting little changes at the faecal level when exposed to probiotic interventions.^63^ It should be taken into account that bile salt absorption occurs in the distal ileum,^40,64^ where microbial density is 10^7^–10^8^ CFU/mL, much lower than in the colon at ∼10^12^ CFU/mL.^65,66^ Therefore, probiotics could significantly impact ileal microbiota (where BA reabsorption occurs), while faecal microbiota remains mostly unchanged due to dilution effects and the changing microbial niche in the colon.^67^ In agreement, no statistic correlation was found between the relative content of faecal microbiota and changes in circulating conjugated BA after probiotic intervention. However, the most prominent reduction in conjugated BA was found in individuals with a relative increase of *Actinocabteria* and *Bacterioidetes* after the intervention with the *L. plantarum* mixture. Interestingly, a recent study of 90 000 gut microbiota clones evidenced 142 with BSH activity, among which phyla *Bacteroidetes* and *Actinobacteria* were highly represented. In the same study, *Actinobacteria* clones were capable of deconjugating all the glyco- and tauro-conjugated BAs tested, while *Bacteroidetes* had specific activity against tauro- but not glyco-conjugated BAs.^68^ Further studies are needed to better define whether changes in BA conjugation after the *L. plantarum* intervention are only dependent on the *Lactobacillus* BSH activity or also related to probiotic-mediated changes in the gut microbiota.

Increasing evidence suggests faecal metabolome as a functional readout of microbial activity.^69^ In this study, analysis of faecal samples by NMR evidenced 29 metabolites consistently detected in all subjects, with an enrichment of amino acids (41% of metabolites) and SCFA (24% metabolites). Four weeks of intervention with *L. plantarum* did not significantly modify the faecal metabolomic profile in healthy subjects. However, most of metabolites showed a coordinated decreasing trend at the end of the intervention period. From our results, we cannot conclude if this is due to increased utilization or decreased production of amino acids and SCFA by the microbiota or results from more complex probiotic-induced host–microbiome interactions.

This mechanistic study has some limitations that warrant consideration, including the single-arm design and the small sample size. However, the structural design simplicity of a single-arm longitudinal design within the framework of a proof-of-concept mechanistic study has allowed to simultaneously investigate the response of a large spectrum of variables to the *L. plantarum* mixture intervention on top of the regular diet. From our results, we cannot exclude some ‘placebo’ effects, but these were minimal since the study was performed in a stable cohort of healthy overweighted individuals, who maintained their dietary and physical activity habits throughout the intervention period, and therefore, spontaneous changes in the participants were not foreseen. However, the results of the study merit new studies with larger sample size and two-arm designs (including a placebo group) to further investigate the molecular processes involving the response of the BA–lipoprotein axis to the probiotic intervention. Besides, to minimize possible bias resulting from inter-individual variability in a small sample size study, we have performed serial measurements (baseline and weekly time points) in each participant coinciding with the end of each probiotic dose period in the dose-escalating design.

In summary, our study provides for the first time evidence in humans that intervention with the BSH-active *L. plantarum* strains KABP011, KABP012, and KABP013 results in a reduction of ApoB100 and ApoB48 plasma levels, significant changes in the profile of the LDL particles with a decrease in small LDL, a significant decrease in LDL susceptibility to be oxidized, and increase in HDL antioxidant capacity. Probiotic-induced effects on the lipoprotein profile went along with a deconjugation of BAs and a decrease in the circulating levels of FGF-19, which may promote *de novo* synthesis of BA in the liver from circulating cholesterol. The present study supports the notion that incorporation of the specific probiotic formulation combining three BSH-active strains (KABP011, KABP012, and KABP013) of *L. plantarum* could be beneficial to control metabolic processes known to be relevant in atherosclerosis.

**Translational perspective**

Intervention with escalating doses of the *Lactiplantibacillus plantarum* strains KABP011, KABP012, and KABP013 during 4 weeks induces changes on BA and lipoprotein profile that are compatible with a pattern of protection against atherosclerosis in overweight subjects. The cardiovascular health-promoting effects of the probiotic include an increase in BA deconjugation in the gut and reduced intestinal reabsorption, a decrease in plasma non-HDLc with a significant reduction in apoB100 and ApoB48 levels, and a decrease in small LDL particles. Importantly, LDL susceptibility to oxidation is reduced, and HDL antioxidant capacity is increased after probiotic intake.

## Supplementary Material

cvae061_Supplementary_Data

## Data Availability

The data underlying this article will be shared on reasonable request to the corresponding author.
