# Supplementary material for: Lactiplantibacillus plantarum strains KABP011, KABP012, and KABP013 modulate bile acids and cholesterol metabolism in humans
Source: Cardiovasc Res. 2024 Mar 25;120(7):708–22. doi: 10.1093/cvr/cvae061 (PMC11135648; doi:10.1093/cvr/cvae061)
Supplement: cvae061_Supplementary_Data [file cvae061_supplementary_data.zip › Padro et al _Suppl Material&Methods __ CVR-2023-0906R1.pdf]

Supplemental Material and Methods

Lactiplantibacillus plantarum strains KABP011, KABP012 and KABP013 modulate Bile Acids and Cholesterol Metabolism in humans

Short Title: ***L. plantarum, bile acid and cholesterol metabolism***

T Padro[1,2,†], V Santisteban[1,3,†], P Huedo[4,5], M Puntes[6], M Aguiló[4], J Espadaler-Mazo[4], L Badimon[1,2,7]*

[†] Both authors have contributed equally

[1] Cardiovascular Program-ICCC, Institut d'Investigació Biomèdica Sant Pau (IIB SANT PAU); Barcelona, Spain
[2]Centro de Investigación Biomédica en Red Cardiovascular (CIBER-CV), Instituto de Salud Carlos III, Madrid, Spain
[3]School of Pharmacy and Food Sciences, University of Barcelona (UB), Barcelona, Spain.
[4]R&D Department, AB-Biotics S.A. (Part of Kaneka Corporation), Barcelona, Spain
[5]Basic Sciences Department, Universitat Internacional de Catalunya, Barcelona, Spain
[6] Medicament Research Center (CIM), Institut d'Investigació Biomèdica Sant Pau (IIB SANT PAU); Barcelona, Spain
[7]UAB-Chair Cardiovascular Research, Barcelona, Spain

Address for corresponding author:
Prof. Lina Badimon
Cardiovascular-Program ICCC
Institut d'Investigació Biomèdica Sant Pau (IIB SANT PAU)
Sant Antoni Mª Claret 167, 08025 Barcelona, Spain
Phone: +34.935565882
Fax: +34.935565559
E-mail: lbadimon@santpau.cat

2. Materials and Methods

2.1. Subjects

Healthy overweight men and women [body mass index (BMI) 25.0-29.9 kg/m^2] between the ages of 25 and 60 years (N=20) were included in the study. Exclusion criteria were reported existing chronic illnesses including cancer, overt hyperlipidemia, diabetes mellitus, hypertension, heart, liver or kidney disease, the use of lipid-lowering drugs, beta-blockers, diuretics or antibiotics, history of CVD, or being in a weight-loss program. To confirm healthy status, all subjects underwent a complete physical examination (including biochemical and hematological variables) conducted by the study physician before entry into the study. Those consuming any dietary supplement with prebiotic / probiotic effects were also excluded.

The study complies with the Declaration of Helsinki. It was approved by the Human Ethical Review Committee of the Hospital de la Santa Creu i Sant Pau in Barcelona (Ref 20/029, March 2020) and was registered in Clinicaltrials.gov with NCT05378230. Informed written consent was obtained from all participants before their inclusion in the study.

2.2. Study design

The intervention trial consisted of a single-center, single-arm, dose-escalation longitudinal study with 4-week intervention period (**Supplemental Figure 1**). Thus, all subjects underwent four sequences of 7 days-treatment with increasing dose of *Lactiplantibacillus plantarum* strains KABP011 (CECT 7527), KABP012 (CECT 7528) and KABP013 (CECT 7529). This *L. plantarum* combination refers to a commercially available product (AB-LIFE®) which consists of vegetable gelatin capsules (hypromellose) containing a mixture in a 1: 1: 1 ratio of the three *L. plantarum* strains. Each capsule contains 1.2x10^9 cfu (colony forming units). Before the initiation of the intervention, individuals were subjected to a two-week run-in period. During the intervention period, men and women received one capsule AB-LIFE® daily during the first 7 days. This dose was selected on basis to a previous randomized-clinical trial study in hypecholesterolmic subjects[1] that showed the efficacy of this dose of Lactobacillus KABP011 (CECT 7527), KABP012 (CECT 7528) and KABP013 (CECT 7529) in reducing plasma cholesterol levels. From the first week on, doses were escalated weekly at the rate of x2, x3, x4 of the initial dose in order to investigate whether a dose-dependent effect could be identified **Supplemental Figure 1.**

Subjects were instructed to maintain their regular dietary habits at levels consistent with maintenance of a stable body weight and to continue their normal pattern of physical activity throughout the study period. Dietary habits, determined by using food frequency questionnaires, were recorded prior to each visit. No changes were reported beyond occasional minor alterations in dietary or physical habits.

Compliance was monitored by regular telephone contact with participants and interviewing them at the end of each intervention period. The participants also recorded whether they had consumed the probiotic product every day in a weekly diary. In addition, at the end of each intervention period a physician assessed any side effects or symptoms possibly associated with the study intervention.

A 50% of the participants had at baseline, LDLc levels under borderline-high range (<130 mg/dL) and 50 % in the borderline-high range (130-159 mg/dL) and high range (160-189 mg/dL levels), according to the ATPIII-guidelines[2] and were defined as High-LDLc group in comparison with those subjects with baseline LDLc <130 mg/dL, defined as Low-LDLc group.

2.3. Biological samples

Twelve-hour fasting blood samples were collected at baseline (day 0) and at the end of each intervention period (days 7, 14, 21, 28) from 8 to 11 hours a.m. Blood samples were collected without anticoagulant or in citrate- and ethylenediamine tetraacetic acid (EDTA)-containing Vacutainer tubes for serum and plasma preparation, respectively. Serum and plasma fractions were separated by centrifugation and stored at -80°C until analysis.

A spot stool sample of each individual was collected in sterile bottle during the 24 h period prior to day 0 (baseline) and day 28 (end of the intervention period), directly stored at -20 ºC up to 24h and thereafter at -80ºC until analysis. A spot stool sample for each individual was directly collected in OMNIgene-GUT tube (microbiota analysis), as described by the provider, within the 24 hours prior to day 0 (day -1) and within the 24 hours prior to day 28 (last day of the study). Samples were stored at room temperature up to 24h and thereafter at -80ºC until analysis, as described by the provider.

2.4. Anthropometric Data, Blood Pressure, Serum Lipid Profile and Other Biochemical Measurements

Anthropometric measurements, blood pressure, serum lipid profile and biochemical measurements were determined at baseline, at days 7, 14, 21 and 28 (see Supplemental Figure 1). Body mass index (BMI) was calculated as weight (Kg)/height $(m)^2$. Waist circumference (WC) was measured between the lowest rib and the iliac crest with the participant standing. Waist-to-hips ratio (WtHR) was calculated as waist circumference in cm divided by hips circumference in cm.

Serum biochemical measurements were performed at the centralized laboratory for analysis of the Hospital de Sant Pau using routine commercially available assays for glucose levels, total bile acids, hepatic and renal markers, C-reactive protein (CRP), and standard serum lipid profile (triglycerides, total cholesterol (TC) and high density lipoprotein (HDL) cholesterol. As there were no cases of hypertriglyceridemia, low density lipoprotein (LDL) and very low-density lipoprotein (VLDL) cholesterol was calculated using the Friedewald equation. Thyroid hormones (thyroid-stimulating hormone [TSH], T3 and T4) were determined by chemiluminescent immunoassays in serum samples.

The serum concentration of lipoprotein A [Lp(a)] was measured using an immunoturbidimetric method on the Abbott Alinity® c platform (Abbott Laboratories). Intra-and inter-assay coefficients of variation, evaluated with Lp(a) Control (Abbott Laboratories) were found to be <7%

2.5. Analysis of Bile Acid levels and profile in serum and feces

Quantitative BA total levels and BA-profile were measured in serum and faecal samples. BA total levels in serum were assessed by colorimetric enzymatic assays and the BA-profile (primary/secondary- and unconjugated/conjugated-BA) was identified by ultra-high performance liquid chromatography – mass spectrometry (UHPLC-MS) after metabolite extraction in methanol as described by Barr et al[3]. Chromatographic separation and MS detection conditions used for the UHPLC-MS method are summarized in Supplemental Figure 2. Identified ion features included unconjugated bile acids (2 primary and 3 secondary in serum, and 2 primary and 5 secondary in feces) and conjugated bile acids including 4 primary (2 tauro- and 2 glyco-conjugated) and 3 secondary (1 tauro- and 2 glyco-congugated) in serum, and 4 primary (2 tauro- and 2 glyco-congugated) and 2 secondary (1 tauro- and 1 glyco-congugated) in feces. Data

were processed using the TargetLynx application manager for MassLynx version 4.1 software (Waters Corp., Milford) as described previously[4].

2.6. Characterization of Lipoprotein particles (number and size), glycoproteins and low molecular weight metabolites by 1H-NMR

Serum samples from baseline, day 14 and day 28 and frozen feces from baseline and day 28 were aliquoted for molecular characterization by high-resolution 1H-NMR spectroscopy. Spectra was recorded on a BrukerAvance III 600 spectrometer, setting at proton frequency of 600.20 MHz (14.1 T), recorded at 310 k and at 300 K for serum and faecal samples respectively.

The lipoprotein profile was obtained by using the Liposcale® test (IVD-CE) as previously described[5] Briefly, the methyl signal from a longitudinal eddy-current delay (LED) pulse spectra was surface fitted with 9 lorentzian functions associated with each lipoprotein subtype: large, medium and small of the VLDL, LDL and HDL. The area of each lorentzian function was related to the lipid concentration of each lipoprotein subtype, and the size of each subtype was calculated from their diffusion coefficient with Stokes-Einstein equation[6, 7]. The lipid concentration units were converted to lipid volume units using common conversion factors [8]. The particle numbers of each lipoprotein subtype were calculated by dividing the lipid volume by the particle volume of a given class[9]. The variation coefficients for particle number were between 2% and 4%, and for the particle sizes were lower than 0.3%

For glycoprotein quantification, we analyzed the region of the same LED 1H-NMR spectrum where the glycoproteins resonate (2.15-1.90 ppm) using several analytical functions according to a previously published procedure[10].

For each function, we determined the total area (proportional to concentration), height, position and bandwidth. The area of GlycA provided the concentration of acetyl groups of protein-bond N-acetylglucosamine and N-acetylgalactosamine, and the area of GlycB those of N- acetylneuraminic acid[11].

Low molecular weight metabolites (LMWM), including short chain fatty acids, aminoacids and sugars among others, from faecal samples were analyzed. One-dimensional ^{1}H pulse experiments were carried out using the nuclear Overhauser effect

spectroscopy (NOESY)-presaturation sequence. The acquired spectra were phased, baseline-corrected and referenced before performing the automatic metabolite profiling of the spectra datased through and adaptation of Dolphin.[12] Several database engines BBioref AMIX database (Bruker), Chenomx and HMDB[13], and literature[14] were used for 1D-resonances assignment and metabolite identification.

2.7. Assays of lipoprotein functionality

2.7.1. Lipoprotein Preparation

LDL (density range 1.019-1.063 g/mL) and HDL (density range 1.063-1.210 g/mL) were obtained from plasma-EDTA from individual samples at baseline and different times (14 and 28 days) during the probiotic intervention by sequential ultracentrifugation, according to the method originally described by Havel et. al[15] and modified by De Juan-Franco et. al[16], as previously described[17]. In addition, LDLs to be used in the total radical trapping potential (TRAP) assay were isolated from a pool of plasma obtained from normolipemic subjects and obtained as described above. LDL and HDL fractions were dialyzed against phosphate buffer saline 1X (PBS 1X) for 24 hours. LDL- and HDL-protein content was determined by the colorimetric assay BCA (Pierce, Thermo Fischer Scientific, Waltham, United States) and adjusted to 100 µg/ml. Samples were left protected of light at 4ºC until analysis. The purity of LDL and HDL was analyzed by electrophoresis, in agarose gels (SAS-MX Lipo 10 kit, Helena Biosciences).

2.7.2. Conjugated dienes assay

Susceptibility of LDL to copper-induced oxidation was assessed by determining the formation of conjugated dienes. Briefly, freshly prepared LDL samples adjusted to 100 µg/mL were analyzed by incubation with a copper (II) sulfate ($CuSO_4 \cdot 5H_2O$) at a final concentration of 5µM. The dynamic change of absorbance was determined (2.5 h at 37ºC) using a SpectraMax 190 Microplate reader (Molecular Devices) by continuously monitoring the formation of conjugated dienes at 234 nm. The total amount of conjugated dienes was calculated as previously described[17].

2.7.3. HDL Antioxidant Potential

The antioxidant capacity of HDL was assessed by the total radical trapping potential (TRAP) test[18]. This method is based on the capacity of HDL to prevent LDL (control LDL)

oxidation. Briefly, samples of LDL ("pool" control) alone or in presence of HDL from each individual were adjusted to 100 µg / ml and incubated with $CuSO_4 \bullet 5H_2O$ (final concentration of 20 µM) for 4h (37ºC). Afterward, 50 µL of EDTA 1mM was added to stop oxidation and samples were incubated with 10 µM DCFH-DA (2',7'-dichlorodihydrofluorescein diacetate) for detection of the oxidation level. Intensity of fluorescence was determined with a Typhoon FLA9500 (GE Healthcare) set at λex=500nm and λem=520nm. As previously described, HDL-antioxidant activity was given as percentage of LDL oxidation level in the presence of HDL respect as of LDL oxidation when incubated in the absence of HDL[17].

2.7.4. HDL Cholesterol Efflux Capacity Assay

The cholesterol efflux capacity of HDL was determined *in vitro* in cholesterol-loaded murine macrophages[19] [20]. Briefly, J774A.1 mouse macrophage were cultured in RPMI 1640 (Roswell Park Memorial Institute medium) containing 10% of heat-inactivated FBS (Fetal bovine serum), 2mM glutamine, 100U/mL penicillin, 100U/mL streptomycin and 10 µg/mL gentamicin and maintained at 37 °C in a humidified atmosphere of 5% CO_2.

To measure cholesterol-efflux, macrophages (1.5×10^5 cells/well seeded in 6-well culture plates) were labelled with [1α ,2α (n)-3H cholesterol] (GE Healthcare, Chicago, United States) at 1 µCi per well during 48 h. Cells were then equilibrated overnight (0.2% bovine serum albumin -BSA-) and thereafter incubated for 4h (37ºC) with RPMI media containing 15% of apolipoprotein B (ApoB) -depleted serum (obtained at baseline and at day 14 and 28 during the probiotic intervention) to promote cholesterol efflux from the [3H] cholesterol-labelled cells. Radioactivity signals were measured in the cells and the cell culture media. Cholesterol efflux is expressed as the percentage of cholesterol released to the medium with respect to the total radioactive cholesterol in the well (radioactivity in the cell + radioactivity in medium)[17].

ApoB-depleted serum was prepared by precipitation with phosphotungstic acid / $MgCl_2$ according to the procedure initially described by Asmman et al[21] and previously reported by us and others [17,19]. The phosphotungstic acid binds to positively charged ApoB containing lipoproteins which are then cross linked by the use of a divalent cation such as Mg to form a precipitate. Briefly, serum samples (150µL) were mixed with 300µL a solution containing 22nM hydrated magnesium chloride and 0.484nM acid phosphotungstic and centrifugated (10,000g) after 10 min incubation (RT) to precipitated ApoB-containing lipoproteins.

Levels of ApoB and ApoA were measured in all serum samples after precipitation of ApoB-containing lipoprotein particles to check sample purity.

2.8. Immunoassays: Apolipoproteins, Fibroblast Grow Factor-19 (FGF-19), glucose-metabolism and inflammatory markers

Serum levels of apolipoprotein B-48 (ApoB48) and B-100 (ApoB100) were measured with commercial sandwich-based ELISA kits (Cloud-Clone Corp, ref SEB883Hu and SEA603Hu, respectively). The detection limits of the assays were 0.9 ng/mL for ApoB-48 and 9.15 ng/mL for ApoB-100.

Adiponectin, insulin, leptin and FGF-19 levels were measured in plasma by commercial ELISAs (Adiponectin and insulin: R&D Systems, ref: DRP300 and DINS00; FGF-19 and leptin: ABCAM; ref ab230943 and ref ab179884) with detection limits of 0.891 ng/mL, 2.15 pmol/L, 1.4 pg/mL and 4.65 pg/mL, respectively.

Plasma level of the interleukins (IL), IL-1β and IL-6 were quantified in serum by commercial ELISAs kits (R&D Systems, ref HSLB00D and D6050, respectively). The detection limits of the assays were 0.063 pg/mL for IL-1β and 0.7 pg/mL for IL-6.

Plasma level of IL-8, IL-12, IL-17A and tumor necrosis factor-alpha (TNF-α) were quantified using the Millipore's MILLIPLEX MAP high sensitivity human cytokine kit (Millipore Corporation, Billerica, MA, USA). The multiplex immunoassay panel was analyzed on a multiplex suspension array system (Luminex 200 IS System). The detection limits for IL-8, IL-12, IL-17 and TNF-α were 0.13, 0.15, 0.33, and 0.16 pg/mL, respectively. All procedures were performed according to the manufacturer's instructions.

2.9 Trimethylamine N-oxide (TMAO)

TMAO analysis was performed as described by Canyelles et al [22] by UHPLC (1290 Infinity II Series) coupled 6490 triple-quadrupole mass spectrometer (QqQ, Agilent Technologies) with an electrospray ion source (LC-ESI-QqQ) working in positive mode. EDTA-plasma samples were extracted in acetonitrile: methanol: water (5:4:1; v:v:v) before analysis.

2.10 Metagenomics methods

DNA was extracted from fecal samples obtained on days 0 and 28 with MoBio's Soil DNA Isolation kit (Qiagen). Bacterial 16S rRNA genes were amplified with primers targeting the V3-V4 region (515F and 806R) for 25 PCR cycles and sequenced with Miseq

(Illumina), obtaining an average of 31,437 demultiplexed reads per sample. Fastq files were quality-filtered and clustered into operational taxonomic unit (OTUs) using the QIIME2 software package [23] and classified using a Bayesian classifier trained on the Silva database v.138[24]. Several diversity metrics were computed: number of OTUs and Shannon index for alpha-diversity, and Bray-Curtiss and Jaccard indexes for beta-diversity. Changes in alpha diversity between day 0 and day 28 were assessed with Wilcoxon test for paired samples, while changes in beta-diversity were assessed by Principal Coordinate Analysis (PCoA) and PERMANOVA. Finally, differential abundance of taxa was assessed by Wilcoxon test for paired samples, using a False Discovery Rate (FDR) threshold of 0.1 [25] to correct for multiplicity of analysis at each taxonomic level.

2.11 Characterization of BSH activity of AB-LIFE Strains

BSH- activity of the L. plantarum strains KABP011, KABP012 and KABP013 was measured in vitro. Shortly, a BA mixture (5mM) containing glycocholic acid (GCA), taurocholic acid (TCA), glycochenodeoxycholic acid (GCDCA) and taurochenodeoxycholic acid (TCDCA) was incubated in the presence of 1x108 CFU bacterial suspension (L. plantarum strains) in 0.5X MRS (De Man, Rogosa and Sharpe agar)-liquid medium for 90 min. BSH-activity was determined by measuring levels of free taurine and glycin at the end of the incubation period using a commercial kits Glycine Assay Kit (ab211100) and Taurine Assay Kit (ab241040) from Abcam (UK), following manufacturer's instructions. Negative control suspensions included MRS 0.5X, MRS 0.5X + BS (5 mM) and MRS 0.5X + bacteria (1x108 CFU), and their absorbance values were subtracted from tested suspensions. Control positive suspensions contained MRS 0.5X + taurine or glycine (5 mM), and their absorbance values were considered as 100% deconjugation activity.

2.12. Statistical analysis

Data are expressed as median and interquartile range [IQR] for the quantitative variable. Individual average changes were calculated as the mean of the changes for each variable and subject at the end of each intervention period compared to baseline.

Effects of the 4-week interventions were evaluated using Friedman test for repeated measures and the Wilcoxon signed-rank non-parametric pair-test. Statistical differences between groups for non-normally distributed continuous variables were analyzed by non-parametric Mann–Whitney tests and their distribution by Chi-squared test. Statistical significance was calculated for the average change (end of each intervention

period compared to baseline) using one-Sample T-Test. Bivariate correlations between continuous variables were assessed by Spearman correlation coefficients and Pearson correlation coefficient. Individual p-values were adjusted by False Discovery Rate (FDR). Statistical analyses were conducted using STATA 15 (College Station, TX, USA) and StatView 5.0.1 software (SAS Institute, Cary, NC, USA) and p-values (two-sides) <0.05 were considered significant.

REFERENCES

1. Fuentes MC, Lajo T, Carrión JM, Cuñé J. A randomized clinical trial evaluating a proprietary mixture of Lactobacillus plantarum strains for lowering cholesterol. Med J Nutrition Metab 2016;9:125–135.

2. Mach F, Baigent C, Catapano AL, Koskina KC, Casula M, Badimon L, Chapman MJ, Backer GG De, Delgado V, Ference BA, Graham IM, Halliday A, Landmesser U, Mihaylova B, Pedersen TR, Riccardi G, Richter DJ, Sabatine MS, Taskinen MR, Tokgozoglu L, Wiklund O, Windecker S, Aboyans V, Collet JP, Dean V, Fitzsimons D, Gale CP, Grobbee D, Halvorsen S, Hindricks G, Iung B, Jüni P, Katus HA, Leclercq C, Lettino M, Lewis BS, Merkely B, Mueller C, Petersen S, Petronio AS, Roffi M, Shlyakhto E, Simpson IA, Sousa-Uva M, Touyz RM, Nibouche D, Zelveian PH, Siostrzonek P, Najafov R, Borne P van de, Pojskic B, Postadzhiyan A, Kypris L, Špinar J, Larsen ML, Eldin HS, Viigimaa M, Strandberg TE, Ferrières J, Agladze R, Laufs U, Rallidis L, Bajnok L, Gudjónsson T, Maher V, Henkin Y, Gulizia MM, Mussagaliyeva A, Bajraktari G, Kerimkulova A, Latkovskis G, Hamoui O, Slapikas R, Visser L, Dingli P, Ivanov V, Boskovic A, Nazzi M, Visseren F, Mitevska I, Retterstøl K, Jankowski P, Fontes-Carvalho R, Gaita D, Ezhov M, Foscoli M, Giga V, Pella D, Fras Z, Perez de Isla L, Hagström E, Lehmann R, Abid L, Ozdogan O, Mitchenko O, Patel RS. 2019 ESC/EAS guidelines for the management of dyslipidaemias: Lipid modification to reduce cardiovascular risk. Atherosclerosis 2019;41:111–188.

3. Barr J, Vázquez-Chantada M, Alonso C, Pérez-Cormenzana M, Mayo R, Galán A, Caballería J, Martín-Duce A, Tran A, Wagner C, Luka Z, Lu SC, Castro A, Marchand-Brustel Y Le, Martínez-Chantar ML, Veyrie N, Clément K, Tordjman J, Gual P, Mato JM. Liquid Chromatography-Mass Spectrometry (LC/MS)-based parallel metabolic profiling of human and mouse model serum reveals putative biomarkers associated with the progression of non-alcoholic fatty liver . J Proteome Res 2010;9:4501.

4. Martínez-Arranz I, Mayo R, Pérez-Cormenzana M, Mincholé I, Salazar L, Alonso

C, Mato JM. Enhancing metabolomics research through data mining. J Proteomics 2015;127:275–288.

5. Mallol R, Amigó N, Rodríguez MA, Heras M, Vinaixa M, Plana N, Rock E, Ribalta J, Yanes O, Masana L, Correig X. Liposcale: a novel advanced lipoprotein test based on 2D diffusion-ordered 1H NMR spectroscopy. J Lipid Res 2015;56:737–746.

6. Mallol R, Rodríguez MA, Heras M, Vinaixa M, Cañ N, Jesús •, Nú B•, Plana R, Masana L, Correig X. Surface fitting of 2D diffusion-edited 1 H NMR spectroscopy data for the characterisation of human plasma lipoproteins. 2011;7:572–582.

7. Johnson CS. Diffusion ordered nuclear magnetic resonance spectroscopy: principles and applications. Prog Nucl Magn Reson Spectrosc 1999;34:203–256.

8. Pintó X, Masana L, Civeira F, Real J, Ibarretxe D, Candas B, Puzo J, Díaz JL, Amigó N, Esteban M, Valdivielso P. Consensus document of an expert group from the Spanish Society of Arteriosclerosis (SEA) on the clinical use of nuclear magnetic resonance to assess lipoprotein metabolism (Liposcale®). Clin Investig Arterioscler 2020;32:219–229.

9. Pintó X, Masana L, Civeira F, Real J, Ibarretxe D, Candas B, Puzo J, Díaz JL, Amigó N, Esteban M, Valdivielso P. Consensus document of an expert group from the Spanish Society of Arteriosclerosis (SEA) on the clinical use of nuclear magnetic resonance to assess lipoprotein metabolism (Liposcale®). Clin Investig Arterioscler 2020;32:219–229.

10. Fuertes-Martín R, Taverner D, Vallvé JC, Paredes S, Masana L, Correig Blanchar X, Amigó Grau N. Characterization of 1H NMR Plasma Glycoproteins as a New Strategy to Identify Inflammatory Patterns in Rheumatoid Arthritis. J Proteome Res 2018;17:3730–3739.

11. Fuertes-Martín R, Moncayo S, Insenser M, Martínez-García MÁ, Luque-Ramírez M, Grau NA, Blanchar XC, Escobar-Morreale HF. Glycoprotein A and B Height-to-Width Ratios as Obesity-Independent Novel Biomarkers of Low-Grade Chronic Inflammation in Women with Polycystic Ovary Syndrome (PCOS). J Proteome Res 2019;18:4038–4045.

12. Gómez J, Brezmes J, Mallol R, Rodríguez MA, Vinaixa M, Salek RM, Correig X, Cañellas N. Dolphin: A tool for automatic targeted metabolite profiling using 1D and 2D 1 H-NMR data. Anal Bioanal Chem 2014;406:7967–7976.

13. Wishart DS, Guo AC, Oler E, Wang F, Anjum A, Peters H, Dizon R, Sayeeda Z, Tian S, Lee BL, Berjanskii M, Mah R, Yamamoto M, Jovel J, Torres-Calzada C, Hiebert-Giesbrecht M, Lui VW, Varshavi D, Varshavi D, Allen D, Arndt D, Khetarpal N, Sivakumaran A, Harford K, Sanford S, Yee K, Cao X, Budinski Z, Liigand J, Zhang L, Zheng J, Mandal R, Karu N, Dambrova M, Schiöth HB, Greiner R, Gautam V. HMDB 5.0: The Human Metabolome Database for 2022. Nucleic Acids Res 2022;50:D622–D631.

14. Vinaixa M, Ángel Rodríguez M, Rull A, Beltrán R, Bladé C, Brezmes J, Cañellas N, Joven J, Correig X. Metabolomic assessment of the effect of dietary cholesterol in the progressive development of fatty liver disease. J Proteome Res 2010;9:2527–2538.

15. HAVEL RJ, EDER HA, BRAGDON JH. The distribution and chemical composition of ultracentrifugally separated lipoproteins in human serum. J Clin Invest 1955;34:1345–1353.

16. Juan-Franco E De, Pérez A, Ribas V, Sánchez-Hernández JA, Blanco-Vaca F, Ordóñez-Llanos J, Sánchez-Quesada JL. Standardization of a Method to Evaluate the Antioxidant Capacity of High-Density Lipoproteins. Int J Biomed Sci 2009;5:402–410.

17. Padro T, Muñoz-García N, Vilahur G, Chagas P, Deyà A, Antonijoan RM, Badimon L. Moderate Beer Intake and Cardiovascular Health in Overweight Individuals. Nutrients 2018;10:1237.

18. Valkonen M, Kuusi T. Spectrophotometric assay for total peroxyl radical-trapping antioxidant potential in human serum. J Lipid Res 1997;38:823–833.

19. Escolà-Gil JC, Lee-Rueckert M, Santos D, Cedó L, Blanco-Vaca F, Julve J. Quantification of in vitro macrophage cholesterol efflux and in vivo macrophage-specific reverse cholesterol transport. Methods in Molecular Biology. Humana Press Inc.; 2015. p211–233.

20. Padró T, Cubedo J, Camino S, Béjar MT, Ben-Aicha S, Mendieta G, Escolà-Gil JC, Escate R, Gutiérrez M, Casani L, Badimon L, Vilahur G. Detrimental Effect of Hypercholesterolemia on High-Density Lipoprotein Particle Remodeling in Pigs. J Am Coll Cardiol 2017;70:165–178.

21. Assmann G, Schriewer H, Schmitz G, Hägele EO. Quantification of high-density-lipoprotein cholesterol by precipitation with phosphotungstic acid/MgCl2. Clin Chem. 1983;29:2026-30.22.

22. Canyelles M, García-Osuna Á, Junza A, Yanes O, Puig N, Ordóñez-Llanos J, Sionis A, Sans-Roselló J, Alquézar-Arbé A, Santos D, Rotllan N, Julve J, Tondo M, Escolà-Gil JC, Blanco-Vaca F. The Capacity of APOB-Depleted Plasma in Inducing ATP-Binding Cassette A1/G1-Mediated Macrophage Cholesterol Efflux-But Not Gut Microbial-Derived Metabolites-Is Independently Associated with Mortality in Patients with ST-Segment Elevation Myocardial Infarction. Biomedicines 2021;9:1336.

23. Bolyen E, Rideout JR, Dillon MR, Bokulich NA, Abnet CC, Al-Ghalith GA, Alexander H, Alm EJ, Arumugam M, Asnicar F, Bai Y, Bisanz JE, Bittinger K, Brejnrod A, Brislawn CJ, Brown CT, Callahan BJ, Caraballo-Rodríguez AM, Chase J, Cope EK, Silva R Da, Diener C, Dorrestein PC, Douglas GM, Durall DM, Duvallet C, Edwardson CF, Ernst M, Estaki M, Fouquier J, Gauglitz JM, Gibbons SM, Gibson DL, Gonzalez A, Gorlick K, Guo J, Hillmann B, Holmes S, Holste H, Huttenhower C, Huttley GA, Janssen S, Jarmusch AK, Jiang L, Kaehler BD, Kang K Bin, Keefe CR, Keim P, Kelley ST, Knights D, Koester I, Kosciolek T, Kreps J, Langille MGI, Lee J, Ley R, Liu YX, Loftfield E, Lozupone C, Maher M, Marotz C, Martin BD, McDonald D, McIver LJ, Melnik A V., Metcalf JL, Morgan SC, Morton JT, Naimey AT, Navas-Molina JA, Nothias LF, Orchanian SB, Pearson T, Peoples SL, Petras D, Preuss ML, Pruesse E, Rasmussen LB, Rivers A, Robeson MS, Rosenthal P, Segata N, Shaffer M, Shiffer A, Sinha R, Song SJ, Spear JR, Swafford AD, Thompson LR, Torres PJ, Trinh P, Tripathi A, Turnbaugh PJ, Ul-Hasan S, Hooft JJJ van der, Vargas F,

Vázquez-Baeza Y, Vogtmann E, Hippel M von, Walters W, Wan Y, Wang M, Warren J, Weber KC, Williamson CHD, Willis AD, Xu ZZ, Zaneveld JR, Zhang Y, Zhu Q, Knight R, Caporaso JG. Reproducible, interactive, scalable and extensible microbiome data science using QIIME 2. Nat Biotechnol 2019 378 2019;37:852–857.

24. Glöckner FO, Yilmaz P, Quast C, Gerken J, Beccati A, Ciuprina A, Bruns G, Yarza P, Peplies J, Westram R, Ludwig W. 25 years of serving the community with ribosomal RNA gene reference databases and tools. J Biotechnol 2017;261:169–176.

25. Benjamini Y, Hochberg Y. Controlling the False Discovery Rate: A Practical and Powerful Approach to Multiple Testing. J R Stat Soc Ser B 1995;57:289–300.
