# Supplementary material for: Lactiplantibacillus plantarum strains KABP011, KABP012, and KABP013 modulate bile acids and cholesterol metabolism in humans
Source: Cardiovasc Res. 2024 Mar 25;120(7):708–22. doi: 10.1093/cvr/cvae061 (PMC11135648; doi:10.1093/cvr/cvae061)
Supplement: cvae061_Supplementary_Data [file cvae061_supplementary_data.zip › Padro et al _Suppl Tables & Suppl Figures__ CVR-2023-0906R1.pdf]

Supplemental Tables and Figures

Lactiplantibacillus plantarum strains KABP011, KABP012 and KABP013 modulate Bile Acids and Cholesterol Metabolism in humans

Short Title: ***L. plantarum, bile acid and cholesterol metabolism***

T Padro[1,2,†], V de Santisteban[1,3,†], P Huedo[4,5], M Puntes[6], M Aguiló[4], J Espadaler-Mazo[4], L Badimon[1,2,7]*

[†] *Both authors have contributed equally*

[1] Cardiovascular Program-ICCC, Institut d'Investigació Biomèdica Sant Pau (IIB SANT PAU); Barcelona, Spain
[2]Centro de Investigación Biomédica en Red Cardiovascular (CIBER-CV), Instituto de Salud Carlos III, Madrid, Spain
[3]School of Pharmacy and Food Sciences, University of Barcelona (UB), Barcelona, Spain.
[4]R&D Department, AB-Biotics S.A. (Part of Kaneka Corporation), Barcelona, Spain
[5]Basic Sciences Department, Universitat Internacional de Catalunya, Barcelona, Spain
[6] Medicament Research Center (CIM), Institut d'Investigació Biomèdica Sant Pau (IIB SANT PAU); Barcelona, Spain
[7] UAB-Chair Cardiovascular Research, Barcelona, Spain

Address for corresponding author:
Prof. Lina Badimon
Cardiovascular-Program ICCC
Institut d'Investigació Biomèdica Sant Pau (IIB SANT PAU)
Sant Antoni Mª Claret 167, 08025 Barcelona, Spain
Phone: +34.935565882
Fax: +34.935565559
E-mail: lbadimon@santpau.cat

Supplemental table 1. Anthropometric, hemodynamic control and biochemical variables during the 4-week intervention period.

	**Baseline**	**Intervention period**				
	**Day 0**	**Day 7**	**Day 14**	**Day 21**	**Day 28**	***p-Value***
Age (years)	**44** [37.5; 47.5]	**44** [37.5; 47.5]	**44** [37.5; 47.5]	**44** [37.5; 47.5]	**44** [37.5; 47.5]	*1.000*
Sex (Women/Men)	10/10	10/10	10/10	10/10	10/10	-
Antropometric parameters						
BMI (Kg/m^2)	**26.5** [25.4; 29.7]	**26.4** [24.8; 29.5]	**26.6** [25.0; 28.9]	**26.4** [25.2; 29.6]	**26.4** [25.1; 29.6]	*0.458*
Weight (Kg)	**73.5** [68.3; 82.3]	**73.5** [68.5; 82.3]	**74.0** [68.5; 81.5]	**74.0** [68.8; 82.5]	**74.5** [68.8; 82.5]	*0.573*
Waist (cm)	**91.0** [82.0; 97.5]	**88.5** [83.0; 96.5]	**86.0** [83.0; 92.0]	**87.0** [84.0; 96.0]	**90.0** [85.0; 95.5]	***0.025***
Hips (cm)	101.0 [99.5; 107.0]	104.5 [100.0; 108.0]	**101.5** [97.0; 107.0]	**103.5** [99.0; 107.0]	**103.0** [99.0; 105.5]	*0.158*
Waist / hips	**0.88** [0.82; 0.92]	**0.84** [0.80; 0.91]	**0.86** [0.81; 0.90]	**0.85** [0.81; 0.90]	**0.86** [0.82; 0.90]	*0.077*
Hemodynamic control						
Systolic blood pressure (mmHg)	**121.0** [111.0; 126.5]	**118.0** [110.0; 122.0]	**116.0** [111.0; 122.5]	**115.5** [110.5; 125.0]	**117.5** [113.0; 122.5]	*0.550*
Diastolic blood pressure (mmHg)	**70.0** [63.5; 77.0]	**72.0** [61.0; 78.5]	**66.0** [59.0; 74.5]	**69.0** [61.0; 75.0]	**68.0** [60.5; 76.5]	*0.536*
Biochemical parameters						
Total BA (µmol/L)	**2.5** [1.8; 5.5]	**2.3** [1.4; 4.6]	**2.0** [1.2; 4.1]	**1.9** [1.4; 4.0]	**2.1** [1.4; 4.0]	*0.055*
AST (UI/L)	**16.7** [15.1; 22.7]	**16.9** [15.5; 22.9]	**17.7** [16.2; 23.3]	**18.1** [15.1; 22.3]	**17.6** [14.5; 21.8]	*0.267*
ALT (UI/L)	**15.5** [10.9; 27.7]	**14.9** [12.9; 25.0]	**18.9** [12.6; 32.6]	**15.4** [11.8; 27.8]	**16.1** [11.7; 28.1]	*0.387*
GGT (UI/L)	**19.2** [15.5; 30.3]	**20.7** [16.4; 27.6]	**21.1** [16.8; 34.7]	**19.6** [15.3; 29.3]	**20.6** [14.8; 28.7]	*0.221*
Glucose (mmol/L)	**4.7** [4.5-4.9]	**4.4** [4.2-4.8]	**4.8** [4.5-4.9]	**4.6** [4.3-4.8]	**4.7** [4.3-4.9]	***0.027***
Creatinine (µmol/L)	**65.8** [54.8; 70.7]	**71.0** [58.9; 78.4]	**60.9** [57.3; 73.3]	**68.6** [58.7; 75.2]	**64.6** [56.9; 75.8]	***0.000***
Urate (µmol/L)	**301.9** [226.0; 385.4]	**317.9** [247.8; 374.3]	**285.6** [248.7; 364.6]	**271.7** [238.2; 387.6]	**293.2** [225.1; 362.5]	*0.512*
Urea (mmol/L)	**4.3** [3.8; 5.1]	**4.4** [3.8; 5.2]	**4.6** [4.0; 5.1]	**4.7** [4.2; 5.1]	**4.5** [3.8; 5.7]	*0.249*
Creatine Kinase (UI/L)	104.0 [62.9-132.0]	108.0 [61.1-143.9]	111.8 [63.8-132.5]	104.2 [67.1-157.1]	95.6 [54.9-134.6]	*0.106*

Values are expressed as median [IQR]. *p-Value*: Friedman non-parametric test for repeated measure. Statistical significance: *p<0.05*. N=20 for all time points. BMI, body max index; BA, bile acids; AST, aspartate transaminase; ALT, alanine transaminase; GGT, gamma-glutamyltransferase.

Supplemental table 2. Serum bile acids analyzed by UHPLC/MS during four-week intervention with probiotic.

Relative intensity / Volume of serum	Baseline<br>Day 0	Intervention period<br>Day 14	Day 28	*p-Value 1*	*p-Value 2 (D0 vs D14)*	*p-Value 3 (D0 vs D28)*
Non-conjugated BA						
Non-conjugated acids	**5.28** [4.16; 7.53]	**4.77** [2.94; 7.56]	**5.79** [3.25; 8.76]	*0.387*	*0.370*	*0.627*
Non-conjugated primary BA	**4.02** [2.70; 5.22]	**3.15** [2.21; 5.65]	**3.68** [2.37; 6.35]	*0.638*	*0.550*	*0.601*
Cholic acid	**3.28** [2.39; 4.16]	**2.92** [1.83; 4.76]	**3.42** [1.81; 4.55]	*0.861*	*0.737*	*0.550*
Chenodeoxycholic acid	**0.46** [0.17; 1.39]	**0.40** [0.24; 1.37]	**0.56** [0.30; 2.06]	*0.951*	*0.478*	*0.970*
Non-conjugated secondary BA	**1.61** [0.96; 2.82]	**1.33** [0.75; 2.16]	**1.24** [0.75; 2.77]	*0.449*	*0.167*	*0.526*
Deoxycholic acid	**1.02** [0.57; 1.77]	**0.71** [0.39; 1.54]	**0.84** [0.41; 1.47]	*0.212*	*0.179*	*0.296*
Ursodeoxycholic + Hyodeoxycholic acid	**0.63** [0.37; 0.92]	**0.55** [0.34; 0.72]	**0.47** [0.31; 1.42]	*0.638*	*0.247*	*0.737*
Conjugated BA						
Conjugated acids	**3.14** [1.89; 4.54]	**2.23** [0.75; 3.24]	**1.89** [1.10; 3.20]	***0.004***	***0.007***	***0.006***
Conjugated primary BA	**2.05** [1.06; 2.87]	**1.43** [0.49; 1.86]	**1.12** [0.72; 1.66]	***0.011***	***0.011***	***0.008***
Taurocholic acid	**0.30** [0.12; 0.49]	**0.15** [0.06; 0.31]	**0.12** [0.06; 0.21]	***0.035***	***0.021***	***0.005***
Glycocholic acid	**0.50** [0.22; 0.78]	**0.31** [0.14; 0.48]	**0.27** [0.21; 0.42]	***0.047***	***0.048***	***0.033***
Taurochenodeoxycholic acid	**0.63** [0.27; 0.93]	**0.39** [0.19; 0.51]	**0.30** [0.17; 0.44]	***0.002***	***0.002***	***0.004***
Glycochenodeoxycholic acid	**0.66** [0.30; 0.83]	**0.47** [0.17; 0.71]	**0.35** [0.20; 0.69]	*0.086*	*0.079*	***0.037***
Conjugated secondary BA	**1.10** [0.59; 2.11]	**0.68** [0.28; 1.35]	**0.58** [0.29; 1.64]	***0.008***	***0.005***	***0.023***
Taurodeoxycholic acid	**0.33** [0.17; 0.71]	**0.15** [0.08; 0.30]	**0.16** [0.10; 0.49]	***0.002***	***0.001***	***0.021***
Glycodeoxycholic acid	**0.53** [0.20; 0.89]	**0.34** [0.14; 0.48]	**0.27** [0.15; 0.78]	*0.086*	***0.004***	*0.053*
Glycoursodeoxycholic acid	**0.16** [0.07; 0.40]	**0.16** [0.05; 0.39]	**0.10** [0.06; 0.27]	*0.729*	*0.490*	*0.412*
Total primary BA	**5.49** [4.75; 8.19]	**4.68** [3.16; 6.80]	**5.47** [3.73; 7.44]	*0.117*	***0.062***	*0.232*
Total secondary BA	**2.44** [1.53; 4.93]	**2.17** [1.28; 3.83]	**2.53** [1.21; 4.14]	***0.043***	***0.012***	*0.126*
Non-conjugated BA/Conjugated BA	**1.89** [1.13; 3.08]	**2.74** [2.00; 4.82]	**4.16** [1.68; 6.18]	***0.035***	*0.057*	***0.033***

Values are expressed as median [IQR]. *p-Value 1*: Friedman non-parametric test for repeated measures; *p-Value 2* and *3*: Wilcoxon signed-rank test. Statistical significance: $p<0.05$. N=20 for all time points. UHPLC/MS, ultra-high performance chromatography coupled to mass spectrometry; BA, bile acids.

Supplemental table 3. Fecal bile acids analyzed by UHPLC/MS during four-week intervention with probiotic.

(Relative intensity / mg dry tissue of feces)	Day 0	Day 28	*p-Value*
Non-conjugated BA			
Non-conjugated acids	**4.31** [2.77; 5.26]	**3.90** [2.84; 5.32]	*0.332*
Primary BA	**1.07** [0.69; 1.67]	**1.22** [0.87; 1.59]	*0.955*
Cholic acid	**0.24** [0.07; 0.58]	**0.25** [0.17; 0.68]	*0.852*
Chenodeoxycholic acid	**0.78** [0.57; 1.09]	**0.72** [0.59; 0.97]	*0.455*
Secondary BA	**3.08** [1.94; 3.65]	**2.45** [1.92; 3.49]	*0.401*
Deoxycholic acid	**0.05** [0.04; 0.34]	**0.10** [0.04; 0.24]	*0.510*
Ursodeoxycholic + Hyodeoxycholic acid	**0.84** [0.49; 0.92]	**0.68** [0.51; 1.11]	*0.737*
Lithocholic acid	**0.92** [0.72; 1.12]	**0.82** [0.65; 0.90]	*0.255*
12-Oxolithocholic acid	**0.86** [0.36; 1.25]	**0.7** **5** [0.27; 0.90]	*0.052*
Conjugated BA			
Conjugated acids	**1.71** [0.71; 5.45]	**1.86** [0.98; 4.37]	*0.941*
Conjugated primary BA	**0.75** [0.35; 3.9]	**1.23** [0.53; 1.79]	*1.000*
Taurocholic acid	**0.24** [0.09; 0.86]	**0.38** [0.20; 0.60]	*0.695*
Glycocholic acid	**0.28** [0.07; 1.44]	**0.29** [0.06; 0.68]	*0.401*
Glycochenodeoxycholic acid	**0.29** [0.09; 1.23]	**0.31** [0.11; 0.76]	*0.970*
Conjugated secondary BA			
Glycodeoxycholic acid	**0.39** [0.21; 0.75]	**0.46** [0.13; 0.92]	*0.737*
* Taurodeoxycholic + taurochenodeoxycholic acid	**0.21** [0.12; 1.03]	**0.24** [0.15; 0.71]	*0.896*
Conjugated BA/ Non-conjugated BA	**2.36** [0.72; 4.54]	**2.17** [1.13; 4.10]	*0.350*

Values are expressed as median [IQR]. *p-Value*: Wilcoxon signed-rank test. Statistical significance: *p<0.05*. N=20 for all time points. UHPLC/MS, ultra-high performance chromatography coupled to mass spectrometry; BA, bile acids. *BA composed of the sum of a primary and secondary BA.

Supplemental table 4. Lipid profile variables analyzed by biochemistry during the 4-week intervention period.

	Baseline	Intervention period				
	**Day 0**	**Day 7**	**Day 14**	**Day 21**	**Day 28**	***p-Value***
Cholesterol (mg/dL)	**202.0** [158.3; 209.4]	**182.3** [165.2; 203.6]	**181.1** [159.8; 207.0]	**188.1** [159.6; 202.2]	**181.9** [156.3; 211.9]	*0.300*
HDLc (mg/dl)	**47.8** [41.4; 56.3]	**47.4** [40.8; 57.5]	**47.2** [40.1; 58.8]	**48.2** [39.7; 55.3]	**50.7** [39.9; 54.8]	*0.813*
Non-HDLc (mg/dL)	**149.0** [109.7; 165.4]	**136.8** [114.9; 157.1]	**130.6** [110.9; 165.6]	**138.7** [109.3; 156.7]	**142.0** [109.5; 161.8]	*0.300*
LDLc (mg/dL)	**128.3** [96.5; 144.6]	**120.2** [96.6; 138.7]	**118.4** [91.6; 144.0]	**116.4** [93.3; 143.0]	**118.5** [92.6; 136.0]	*0.445*
VLDLc (mg/dL)	**15.1** [9.4-; 25.8]	**13.3** [10.1; 21.5]	**13.9** [10.2; 23.3]	**14.4** [10.6; 23.1]	**17.6** [9.9; 25.9]	*0.119*
TG (mg/dL)	**76.6** [47.4; 130.6]	**67.3** [50.9; 108.9]	**70.4** [51.4; 117.8]	**72.6** [53.6; 116.9]	**89.0** [50.0; 131.1]	*0.119*
Lp(a) (mg/dL)	**15.2** [8.1; 37.9]	-	**17.8** [8.7; 37.6]	-	**16.9** [8.6; 36.0]	*0.502*

Values are expressed as median [IQR]. *p-Value*: Friedman non-parametric test for repeated measure. Statistical significance: $p<0.05$. N=20 for all time points. HDLc, high-density lipoprotein cholesterol; LDLc, low-density lipoprotein cholesterol; VLDLc, very-low density lipoprotein cholesterol; TG, triglycerides; Lp(a), lipoprotein A.

Supplemental table 5. (A) BMI and lipid profile variables, **(B)** serum bile acids analyzed by UHPLC/MS and **(C)** FGF-19 analyzed by ELISA at baseline according LDLc levels.

A

	Low-LDLc	High-LDLc	*p-Value*
BMI (Kg/m^2)	**25.85** [25.10; 29.70]	**26.8** [25.70; 29.60]	*0.406*
HDLc (mg/dL)	**50.5** [44.5; 56.1]	**44.7** [40.6; 56.5]	*0.678*
Non-HDLc (mg/dL)	**109.7** [102.9; 138.2]	**165.2** [150.2; 176.9]	***0.001***
Remnant cholesterol (mg/dL)	**11.2** [8.6; 29.2]	**17.1** [12.8; 21.5]	*0.473*
VLDLc (mg/dL)	**11.2** [8.6; 29.2]	**17.1** [12.8; 21.5]	*0.520*
ApoB100(µg/mL)	**329.4** [228.4; 471.1]	**650.6** [559.0; 900.2]	***0.003***
ApoB48 (µg/mL)	**6.7** [2.9; 9.7]	**14.9** [11.1; 20.3]	***0.003***
TG (mg/dL)	**56.7** [43.4; 147.9]	**86.4** [64.7; 108.9]	*0.520*
Lp(a) (mg/dL)	**14.0** [3.1; 37.4]	**23.3** [8.2; 38.4]	*0.289*

B

(RI/Vol.Ser)	Low-LDLc	High- LDLc	*p-Value*
TBA	**7.33** [6.74; 9.12]	**11.43** [7.47; 1.34]	*0.131*
NC BA	**4.69** [3.92; 6.11]	**6.78** [4.74; 8.14]	*0.227*
NC P BA	**3.73** [2.36; 4.49]	**4.70** [3.03; 5.44]	*0.364*
NC S BA	**1.26** [0.86; 2.20]	**2.20** [1.05; 3.42]	*0.364*
C BA	**2.26** [1.53; 3.47]	**3.97** [2.70; 5.00]	*0.082*
C P BA	**1.27** [0.92; 2.20]	**2.73** [1.99; 3.17]	*0.082*
C S BA	**0.95** [0.36; 1.46]	**1.30** [0.62; 2.12]	*0.326*

C

	Low-LDLc	High- LDLc	*P-value*
**FGF-19** (pg/ml)	**164.40** [128.47; 360.93]	**273.96** [173.03; 370.00]	*0.369*

Values are expressed as median [IQR]. Baseline LDLc levels of Low-LDLc and High-LDLc subgroup were 96.5 [80.9; 119.4] mg/dL and 144.6 [139.6; 152.4] mg/dL. Subject nº3 with outlayers levels of FGF-19 at day 14 was excluded of the analysis in panel C. *p- Value*: Mann–Whitney U test. Statistical significance: $p<0.05$. N=20 for all time points in each panel. UHPLC-MS, ultra-performance liquid chromatography-tandem mass spectrometry; LDLc, low-density lipoprotein cholesterol; RI, relative intensity; Vol. Ser, volume of serum; TBA, total bile acids, NC BA, non-conjugated bile acids; NC P BA, non-conjugated primary bile acids; NC S BA, non-conjugated secondary bile acids; C BA, conjugated bile acids; C P BA, conjugated primary bile acids; C S BA , conjugated secondary bile acids; FGF, fibroblast growth factor.

Supplemental table 6. **(A)** Serum bile acids analyzed by UHPLC/MS and **(B)** FGF-19 analyzed by ELISA of Low-LDLc and High-LDLc subgroups during the 4-week intervention period.

A

**RI/Vol.Ser**	**Day 0**	**Day 14**	**Day 28**	***p-Value***
Low-LDLc				
TBA	**7.33** [6.74; 9.12]	**5.44** [3.46; 6.80]	**7.60** [4.27; 9.44]	*0.150*
NC BA	**4.69** [3.92; 6.11]	**3.50** [2.78; 4.68]	**5.10** [3.36; 6.58]	*0.273*
NC P BA	**3.73** [2.36; 4.49]	**2.73** [2.24; 3.31]	**3.68** [2.31; 4.84]	*0.273*
NC S BA	**1.26** [0.86; 2.20]	**1.13** [0.53; 1.33]	**1.15** [0.69; 2.04]	*0.067*
C BA	**2.26** [1.53; 3.47]	**0.78** [0.65; 2.28]	**1.51** [0.74; 1.97]	*0.150*
C P BA	**1.27** [0.92; 2.20]	**0.56** [0.45; 1.48]	**0.88** [0.59; 1.40]	*0.123*
C S BA	**0.95** [0.36; 1.46]	**0.32** [0.27; 0.83]	**0.52** [0.22; 0.80]	*0.273*
High-LDLc				
TBA	**11.43** [7.47; 1.34]	**9.60** [7.79; 15.85]	**8.50** [6.30; 16.49]	*0.407*
NC BA	**6.78** [4.74; 8.14]	**6.76** [5.34; 10.23]	**6.36** [3.14; 14.21]	*0.905*
NC P BA	**4.70** [3.03; 5.44]	**5.11** [2.17; 7.84]	**4.41** [2.44; 10.47]	*0.905*
NC S BA	**2.20** [1.05; 3.42]	**1.91** [0.81; 3.48]	**2.21** [0.87; 3.93]	*0.497*
C BA	**3.97** [2.70; 5.00]	**2.69** [2.19; 5.10]	**2.14** [1.71; 4.41]	***0.020***
C P BA	**2.73** [1.99; 3.17]	**1.79** [1.42; 3.07]	**1.38** [0.94; 2.11]	***0.014***
C S BA	**1.30** [0.62; 2.12]	**1.03** [0.49; 1.75]	**0.94** [0.50; 1.89]	***0.020***

B

**FGF-19**(pg/ml)	**Day 0**	**Day 14**	**Day 28**	***p-Value***
Low-LDLc	**164.40** [128.47; 360.93]	**178.21** [122.82; 235.81]	**116.69** [76.61; 202.20]	***0.045***
High-LDLc	**273.96** [173.03; 370.00]	**257.55** [149.93; 427.93]	**119.34** [99.07 ; 275.79]	*0.097*

Values are expressed as median [IQR] Baseline LDLc levels of Low-LDLc and High-LDLc subgroup were 96.5 [80.9; 119.4] mg/dL and 144.6 [139.6; 152.4] mg/dL. Subject nº3 with outlayers levels of FGF-19 at day 14 was excluded of the analysis in panel B. *p-Value*: Friedman non-parametric test for repeated measure. Statistical significance: *p<0.05*. N=20 for all time points in each panel. UHPLC-MS, ultra-performance liquid chromatography-tandem mass spectrometry; RI, Relative intensity; Vol.Ser, volume of serum; LDLc, low-density lipoprotein cholesterol; TBA, total bile acids, NC BA, non-conjugated bile acids; NC P BA, non-conjugated primary bile acids; NC S BA, non-conjugated secondary bile acids; C BA, conjugated bile acids; C P BA, conjugated primary bile acids; C S BA, conjugated secondary bile acids; FGF, Fibroblast growth factor.

Supplemental table 7. **(A)** Diameter of circulating lipoproteins and **(B)** concentration of LDL lipoproteins analyzed by MNR during four-week intervention with probiotic.

A

	Baseline	Intervention period		
	**Day 0**	**Day 14**	**Day 28**	***p-Value***
Diameter (nm)				
HDL	**8.23** [8.21; 8.30]	**8.23** [8.21; 8.28]	**8.25** [8.21; 8.28]	*0.886*
LDL	**20.96** [20.71; 21.10]	**21.00** [20.87; 21.17]	**21.12** [20.94; 21.23]	***0.036***
VLDL	**42.14** [41.91; 42.33]	**42.20** [41.97; 42.35]	**42.12** [41.90; 42.28]	*0.819*

B

	Baseline	Intervention period		
	**Day 0**	**Day 14**	**Day 28**	***p-Value***
Particles number (nmol/L)				
LDL	**1449.7** [1201.9; 1641.9]	**1365.3** [1223.5; 1589.4]	**1450.6** [1189.1; 1566.4]	*0.350*
Large LDL	**200.8** [179.2; 215.2]	**199.9** [188.6; 208.8]	**201.0** [188.5; 222.0]	*0.387*
Medium LDL	**428.2** [323.9; 512.3]	**417.8** [354.9; 534.0]	**457.0** [340.0; 520.1]	*0.638*
Small LDL	**759.5** [671.9; 885.2]	**740.1** [656.9; 861.4]	**725.2** [649.3; 853.8]	***0.047***

Values are expressed as median [IQR]. *p-Value*: Friedman non-parametric test for repeated measures. Statistical significance: $p<0.05$. N=20 for all time points in each panel. MNR, magnetic nuclear resonance; HDL, high-density lipoprotein; LDL, low-density lipoprotein; VLDL, very low-density lipoprotein.

Supplemental Table 8. Changes of inflammation markers levels analyzed by ELISA at baseline according to baseline of inflammation markers levels.

		Baseline level < Cut off			Baseline level >Cut off			
	**Cut off**	**Day 0**	**Day 28-Day 0**	***p-Value 1***	**Day 0**	**Day 28-Day 0**	***p-Value 1***	***p-Value 2***
IL-1β (pg/mL)	**0.13**	**0.09** ± 0.02	**0.07** ± 0.09	***0.041***	**0.18** ± 0.06	**0.01** ± 0.08	*0.953*	*0.119*
IL-6 (pg/mL)	**0.67**	**0.22** ± 0.24	**0.26** ± 0.58	*0.428*	**1.35** ± 0.57	**-0.78** ± 0.41	***0.012***	***0.001***
IL-8 (pg/mL)	**10.57**	**0.76** ± 0.35	**-0.13** ± 0.35	*0.084*	**28.80** ± 20.58	**6.89** ± 23.43	*1.000*	*0.582*
IL-12 (pg/mL)	**1.69**	**1.03** ± 0.29	**0.04** ± 0.29	*0.532*	**3.65** ± 1.55	**-0.83** ± 1.25	*0.138*	***0.033***
IL-17 (pg/mL)	**5.74**	**3.50** ± 0. 82	**0.38** ± 2.08	*0.650*	**12.44** ± 6.97	**-1.92** ± 3.56	*0.225*	*0.206*
CRP (mg/L)	**1.61**	**0 .77** ± 0. 42	**0.29** ± 0.58	*0.054*	**3.17** ± 1.37	**-0.59** ± 1.01	*0.176*	***0.030***
TNF-α (pg/mL)	**4.34**	**2.75** ± 0.82	**0.10** ± 0.61	*0.754*	**6.72** ± 1.81	**-0.23** ± 3.94	*0.327*	*0.190*
GlycA (µmol/L)	**755.68**	**639.25** ± 67.88	**19.26** ± 53.98	*0.311*	**971.90** ± 179.59	**-54.74** ± 88.51	*0.128*	***0.043***
GlycB (µmol/L)	**351.66**	**321.88** ± 22.72	**-5.11** ± 19.33	*0.575*	**371.51** ± 15.32	**-1.93** ± 19.06	*0.638*	*0.877*

Values are expressed as mean ± standard deviation. Cut-off is the baseline mean value. *p-Value 1*: Wilcoxon signed-rank test. *p-Value 2*: Mann–Whitney U test. Statistical significance: *p<0.05*. N=20 for all time points. ELISA, enzyme-linked immunosorbent assay; IL, Interleukine; CRP, C-reactive protein; TNF, Tumor necrosis factor; Glyc, Glycoprotein.

Supplemental table 9. Thyroid hormones, and TMAO analyzed by chemiluminescent immunoassays and UHPLC/MS respectively during four-week intervention with probiotic.

	**Baseline**	**Intervention period**		
	**Day 0**	**Day 14**	**Day 28**	***p-Value***
Thyroid hormones				
TSH (mUI/L)	**1.4** [0.8; 2.1]	**1.2** [0.9; 2.4]	**1.5** [0.9; 1.9]	*0.549*
T4 (pmol/L)	**11.0** [10.3; 11.8]	**11.0** [10.7; 11.8]	**11.1** [10.4; 11.9]	*0.534*
T3 (pmol/L)	**4.4** [4.1; 4.7]	**4.4** [4.1; 4.8]	**4.5** [4.2; 5.0]	*0.861*
				P-value
TMAO	**2.4** [1.4; 3.6]	-	**3.5** [2.1; 6.1]	*0.126*

Values are expressed as median [IQR]. Thyroid hormones P-value: Friedman non-parametric test for repeated measures. p-Value: Wilcoxon signed-rank test. Statistical significance: $p<0.05$. N=20 for all time points. TMAO, trimethylamine N-oxide UHPLC/MS, ultra-high performance chromatography coupled to mass spectrometry; TSH, thyroid-stimulating hormone; T4, thyroxine; T3, triiodothyronine.

Supplemental table 10. SCFA analyzed by NMR during four-week intervention with probiotic.

	Day 0	Day 28	Δ (Day 28-Day 0)	*p-Value*
Serum (μmol/L)				
Acetate	**26.3** [19.1; 29.8]	**23.4** [17.5; 30.9]	**-2.22** [-6.72; 4.54]	*0.412*
Faeces (mmol/Kg faecal tissue)				
Acetate	**75.65** [48.12; 109.79]	**63.39** [49.00; 89.72]	-**7.41** [-37.55; 28.78]	*0.550*
Butyrate	**12.54** [4.23; 18.93]	**9.90** [6.86; 17.95]	-**1.29** [-7.85; 6.87]	*0.904*
Propionate	**12.30** [7.24; 19.58]	**10.94** [7.61; 16.16]	**0.39** [-7.88; 6.51]	*0.911*
Formate	**0.22** [0.17; 0.26]	**0.22** [0.18; 0.27]	-**0.02** [-0.05; 0.08]	*0.970*
Isobutyrate	**0.32** [0.13; 0.95]	**0.37** [0.12; 0.41]	-**0.19** [-0.60; 0.19]	*0.093*
Valerate	**2.05** [0.95; 2.86]	**1.39** [1.02 ; 2.45]	**0.01** [-0.83; 0.62]	*0.709*
Caprylate*	**0.43** [0.01; 0.98]	**0.33** [0.22; 0.66]	-**0.20** [-0.72; 0.40]	*0.272*

Values are expressed as median [IQR]. *p-Value*: Wilcoxon signed-rank test. Statistical significance: $p<0.05$. N=20 for all time points except for caprylate (Day 0 = 1 subject levels under detected limit; Day 28 = 5 subjects levels under detected limit) and butyrate (Day 28 = 1 subject levels under detected limit), SCFA, short chain fatty acids; NMR, nuclear magnetic resonance. *Caprylate is a saturated medium chain fatty acid.

Supplemental table 11. Fecal LMWM analyzed by NMR during four-week intervention with probiotic.

(mmol/Kg faecal tissue)	Day 0	N	Day 28	N	Δ (Day 28-Day 0)	N	*p-Value*
Amino acids							
Alanine	**5.95** [3.46; 10.02]	19	**4.59** [3.05; 6.66]	20	**-1.46** [-4.74; 0.94]	19	*0.126*
Alloisoleucine	**0.25** [0.14; 0.47]	18	**0.20** [0.16; 0.46]	19	**-0.01** [-0.11; 0.07]	17	*0.723*
Aspartate	**0.58** [0.36; 0.90]	20	**0.61** [0.39; 0.91]	20	**0.02** [-0.33; 0.39]	20	*0.794*
Glutamate	**18.42** [10.83; 28.89]	20	**14.81** [11.44; 20.72]	20	**-2.20** [-12.13; 3.76]	20	*0.204*
Glycine	**1.63** [0.82; 2.22]	19	**1.00** [0.49; 1.47]	20	**-0.69** [-1.39; 0.16]	19	***0.014***
Isoleucine	**1.67** [0.65; 2.96]	19	**1.20** [0.82; 2.00]	20	**-0.68** [-1.41; 0.40]	19	*0.126*
Leucine	**3.66** [2.27; 7.25]	20	**2.90** [2.30; 5.65]	20	**-0.83** [-2.09; 1.37]	20	*0.412*
Methionine	**1.16** [0.59; 2.42]	20	**1.01** [0.74; 1.77]	20	**-0.27** [-0.91; 0.45]	20	*0.279*
Phenylalanine	**1.19** [0.70; 2.39]	20	**0.90** [0.74; 1.62]	20	**-0.40** [-0.77; 0.52]	20	*0.332*
Sarcosine	**0.10** [0.08; 0.14]	20	**0.07** [0.04; 0.11]	20	**-0.04** [-0.07; 0.00]	20	***0.025***
Tyrosine	**1.44** [0.92; 2.69]	20	**1.05** [0.80; 1.77]	20	**-0.51** [-0.85; 0.26]	20	*0.135*
Valine	**2.14** [1.14; 3.67]	19	**1.54** [1.05; 2.89]	20	**-0.63** [-1.24; 0.29]	19	*0.159*
Glucose metablism							
Lactate	**0.73** [0.67; 1.01]	20	**0.73** [0.59; 0.86]	19	**-0.06** [-0.36; 0.18]	19	*0.421*
Glucose	**3.39** [0.59; 6.75]	20	**1.91** [0.95; 5.58]	20	**0.04** [-2.56; 1.94]	20	*0.852*
Succinate	**2.12** [0.81; 3.28]	20	**2.00** [1.10; 4.55]	20	**0.47** [-1.27; 2.25]	20	*0.247*
Pyrimidine and purine metablism							
Uracil	**0.79** [0.23; 1.58]	19	**0.56** [0.32; 0.73]	20	**-0.21** [-0.77; 0.39]	19	*0.159*
Hypoxanthine	**0.80** [0.40; 1.17]	19	**0.47** [0.25; 0.66]	20	**-0.14** [-0.73; 0.06]	19	*0.059*
Microbial metabolism							
Dimethylamine	**0.09** [0.04; 0.14]	20	**0.08** [0.03; 0.13]	20	**-0.01** [-0.05; 0.06]	20	*0.823*
Methylamine	**0.23** [0.05; 0.34]	20	**0.15** [0.05; 0.32]	19	**-0.05** [-0.27; 0.08]	19	*0.184*
Others							
3-Hydroxyisobutyrate	**4.07** [2.04; 6.58]	20	**3.21** [2.20; 4.54]	20	**-0.81** [-3.92; 1.50]	20	*0.279*
Phenylacetate	**0.88** [0.37; 1.26]	18	**0.38** [0.22; 0.76]	18	**0.38** [0.22; 0.76]	17	*0.177*
Glycerol	**0.77** [0.36; 6.00]	20	**0.87** [0.37; 3.63]	20	**-0.36** [-1.45; 0.24]	20	*0.145*

Values are expressed as median [IQR]. *p-Value*: Wilcoxon signed-rank test. Statistical significance: $p<0.05$. 3-Hydroxyisobutyrate and Phenylacetate are valine and phenylalanine catabolism metabolites respectively and glycerol is an alcohol. LMWM, low molecular weight metabolites; NMR, nuclear magnetic resonance.

Supplemental table 12. Relative abundance of bacterial philums and classes in fecal samples during four-week intervention with probiotic.

Dominium	***Philum***	***Class***	**Day 0**	**N**	**Day 28**	**N**	***p-Value***
Bacteria	***Bacteroidota***	*Bacteroidia*	**43.55** [39.17 49.85]	20	**46.61** [41.19; 49.78]	**20**	*0.478*
	Firmicutes		**41.17** [37.22; 46.55]	20	**39.53** [33.67; 43.67]	**20**	*0.126*
		Clostridia	**34.48** [30.64; 38.46)	**20**	**31.28** [26.91; 35.71]	**20**	*0.079*
		Bacilli	**3.18** [2.12; 6.18)	**20**	**4.74** [2.47; 6.49]	**20**	*0.218*
		Negativicutes	**1.57** [1.32; 4.06	**20**	**1.99** [1.38; 3.71]	**20**	*0.881*
		Incertae Sedis	**0.06** [0.06; 0.06]	1	**0.07** [0.07; 0.07]	1	*0.317*
	Proteobacteria		**6.64** [3.86; 14.51]	20	**8.66** [3.72; 16.19]	**20**	*0.433*
		Gammaproteobacteria	**3.75** [2.96; 6.33]	**20**	**6.95** [3.40; 9.35]	**20**	*0.073*
		Alphaproteobacteria	**6.81** [1.90; 9.08]	9	**3.81** [2.43; 8.06]	9	*0.263*
	Actinobacteriota		**0.83** [0.22; 1.80]	18	**0.79** [0.23; 1.72]	18	*0.981*
		Actinobacteria	**1.04** [0.46; 1.62]	15	**0.94** [0.20; 1.83]	14	*0.285*
		Coriobacteriia	**0.18** [0.09; 0.23]	10	**0.29** [0.15; 0.49]	12	*0.249*
	Verrucomicrobiota		**1.12** [0.85; 5.57]	14	**2.81** [0.98; 5.50]	14	*0.530*
		Verrucomicrobiae	**0.93** [0.64; 6.17]	12	**2.99** [1.61; 5.26]	10	*0.799*
		Lentisphaeria	**0.32** [0.22; 0.84]	11	**0.84** [0.36; 0.97]	13	*0.285*
	Desulfobacterota	*Desulfovibrionia*	**0.87** [0.52; 1.19]	**20**	**1.39** [0.83; 1.72	19	*0.004*
	Cyanobacteria		**0.62** [0.37; 2.87]	7	**0.99** [0.15; 2.14]	6	*0.465*
		Vampirivibrionia	**0.62** [0.43; 2.13]	7	**1.94** [0.95; 2.48]	4	*0.465*
		Cyanobacteriia	**0.13** [0.13; 0.13]	1	**0.17** [0.09; 0.24]	2	-
	Fusobacteriota	*Fusobacteriia*	**1.78** [1.30; 2.26]	2	**0.43** [0.19; 0.67]	2	*0.317*
	Synergistota	*Synergistia*	**0.09** [0.09; 0.09]	2	**0.06** [0.06; 0.06]	1	-
	Elusimicrobiota	*Elusimicrobia*	**1.49** [1.49; 1.49]	1	**2.11** [2.11; 2.11]	1	*0.317*
	Spirochaetota	*Spirochaetia*	**0.25** [0.25; 0.25]	1	-	0	-
	Patescibacteria	*Saccharimonadia*	**0.03** [0.03; 0.03]	1	-	0	-
	Campilobacterota	*Campylobacteria*	**0.02** [0.02; 0.02]	1	-	0	-
Archaea	***Euryarchaeota***	*Methanobacteria*	**0.16** [0.11; 0.20]	8	**0.19** [0.16; 0.23]	9	*0.686*
	Thermoplasmatota	*Thermoplasmata*	**0.46** [0.16; 1.40]	5	**1.41** [0.64; 2.45]	4	*0.715*
Unassigned	*Unassigned*	*Unassigned*	**1.07** [0.56; 1.59]	2	-	0	-

Values are expressed as median [IQR]. *p-Value*: Wilcoxon signed-rank test. Statistical significance: $p<0.05$.

Supplemental Table 13. Spearman correlations between baseline microbial phyla RA and changes (Δ) of BA **(A)** and Apo B **(B)**.

A

	T BA		C BA		N C BA/ C BA	
	**Rho**	***p-Value***	**Rho**	***p-Value***	**Rho**	***p-Value***
Serum BA						
Bacteroidota	0.123	*0.605*	0.286	*0.222*	-0.168	*0.478*
Firmicutes	-0.227	*0.336*	-0.217	*0.359*	0.044	*0.855*
Proteobacteria	0.248	*0.292*	0.036	*0.880*	0.221	*0.349*
Actinobacteriota	-0.333	*0.177*	-0.092	*0.717*	-0.009	*0.971*
Desulfobacterota	0.017	*0.945*	0.247	*0.295*	-0.319	*0.171*
Faecal BA						
Bacteroidota	0.053	*0.826*	-0.053	*0.826*	0.155	*0.514*
Firmicutes	-0.286	*0.222*	-0.143	*0.548*	-0.162	*0.494*
Proteobacteria	-0.047	*0.845*	0.084	*0.724*	-0.038	*0.875*
Actinobacteriota	0.356	*0.147*	0.424	*0.079*	-0.317	*0.200*
Desulfobacterota	-0.033	*0.890*	-0.196	*0.409*	0.397	*0.083*

B

	ApoB48		ApoB100	
	**Rho**	***p-Value***	**Rho**	***p-Value***
Bacteroidota	0.029	*0.905*	-0.272	*0.246*
Firmicutes	0.244	*0.301*	0.223	*0.346*
Proteobacteria	-0.098	*0.682*	-0.104	*0.663*
Actinobacteriota	-0.040	*0.874*	-0.016	*0.951*
Desulfobacterota	-0.119	*0.618*	-0.227	*0.336*

p-Value: Spearman's rank correlation coefficient tests. Statistical significance: $p<0.05$. N=20 in each panel except for correlations with *Actinobacteriota* (3 subjects levels under detected limit at some time point). BA, bile acids; T BA, total bile acids; C BA, conjugated bile acids; N C BA, non conjugated bile acids; Apo, apolipoprotein. Δ, change at day 28 respect baseline.

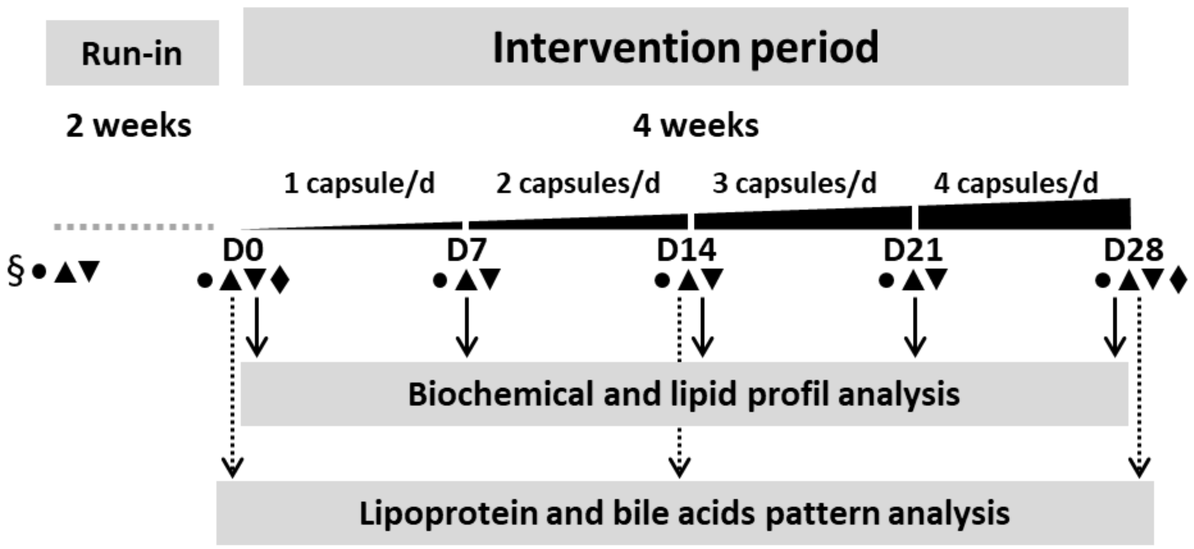

§ Recruitment and analytics for inclusion
● Medical visit, exercise survey, dietary habits
▲ Anthropometric measures
▼ Blood withdraw
⧫ Collection of stool sample

Suplemental Figure 1. Flow diagram describing the study design. D, day.

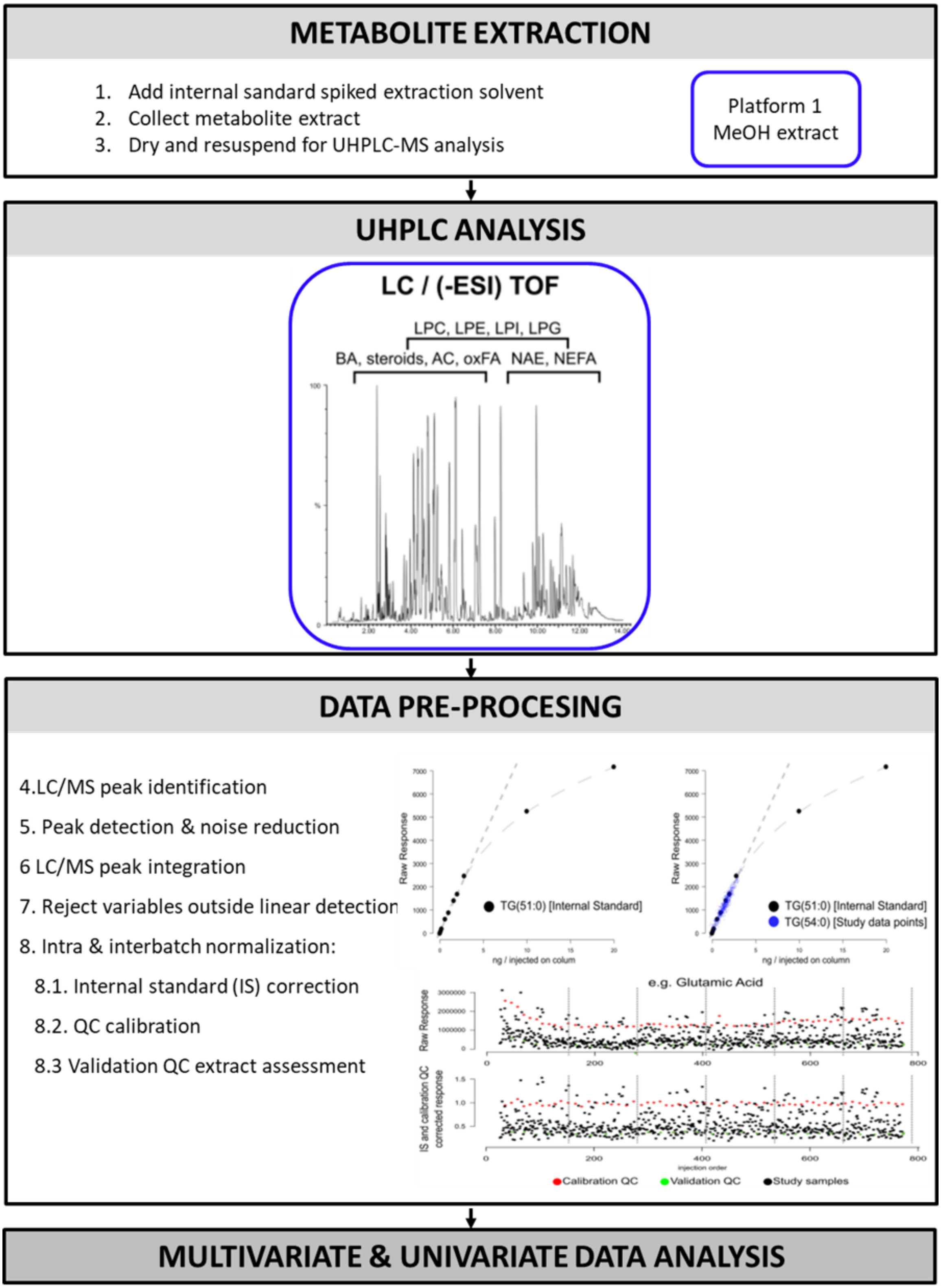

Supplemental Figure 2. Chromatographic separation and MS detection conditions used for the UHPLC-MS method. UHPLC-MS, Ultra-high performance liquid chromatography tandem mass spectrometry; MeOH, methyl hydroxide; LC, liquid chromatography; ESI, electrospray Ionization; TOF, time-of-flight; LPC, lysophosphatidylcholines; LPE, lysophosphatidylethanolamines; LPI, Lysophosphatidylinositol; LPG, lysophosphatidylglycerol; BA, bile acids; oxFA, oxidized fatty acids; NAE, N-acyl ethanolamines; NEFA, non-esterified fatty acid; QC, quality control.

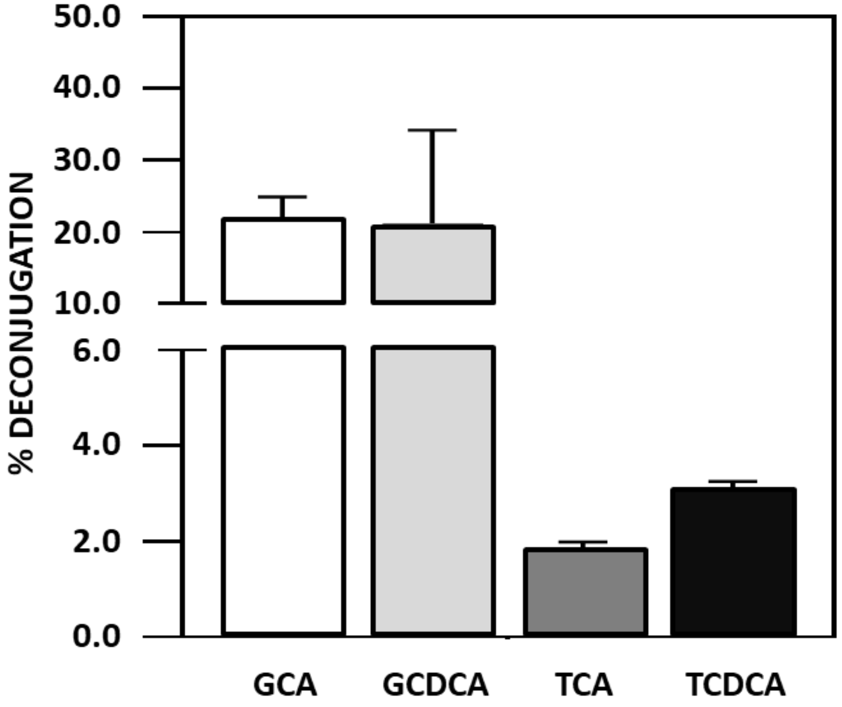

Supplemental Figure 3. Overall percentage of deconjugation activity of GCA, GCDCA, TCA and TCDCA by AB-LIFE formulation respect . The absorbance values of control positive suspensions [taurine or glycine (5 mM)+ MRS 0.5x] were considered as 100% of deconjugation activity. GCA, glycocholic acid; GCDCA, glycochenodeoxycholic; TCA, taurocholic acid; TCDCA, taurochenodeoxycholic; MRS, De Man, Rogosa and Sharpe agar-liquid medium. N=3 (each in triplicates).

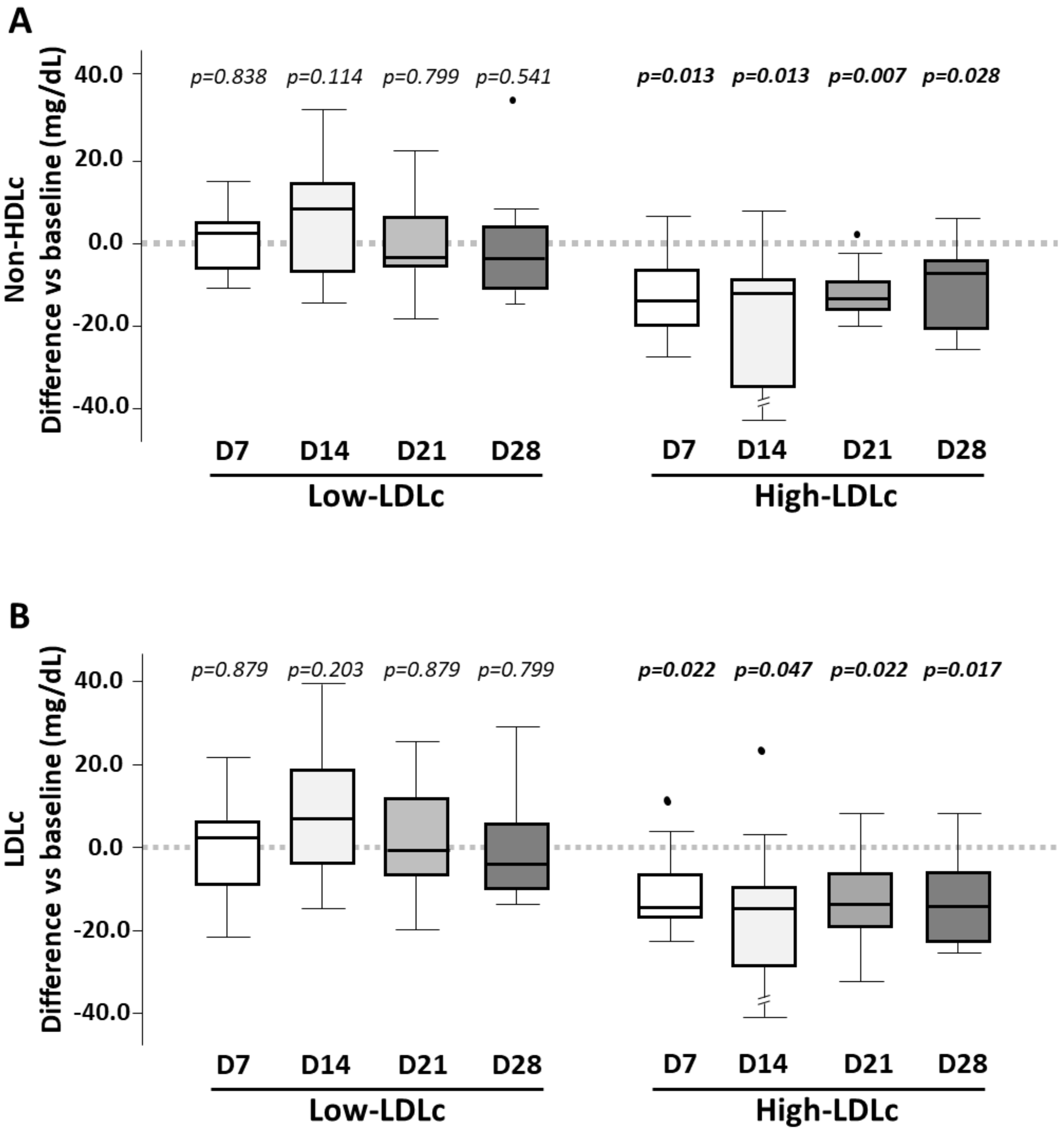

Supplemental Figure 4. Effect of the 4-weeks probiotic intervention in lipid variables. **(A)** Box plots representing difference respect baseline of serum non-HDLc and **(B)** of serum LDLc due to intervention with probiotic in subjects with low and high baseline LDLc levels. *p-Value*: Wilcoxon signed-rank test. Statistical significance: $p < 0.05$. N=20 for all time points in each panel. HDLc, high-density lipoprotein cholesterol; LDLc, low-density lipoprotein cholesterol; D, day.

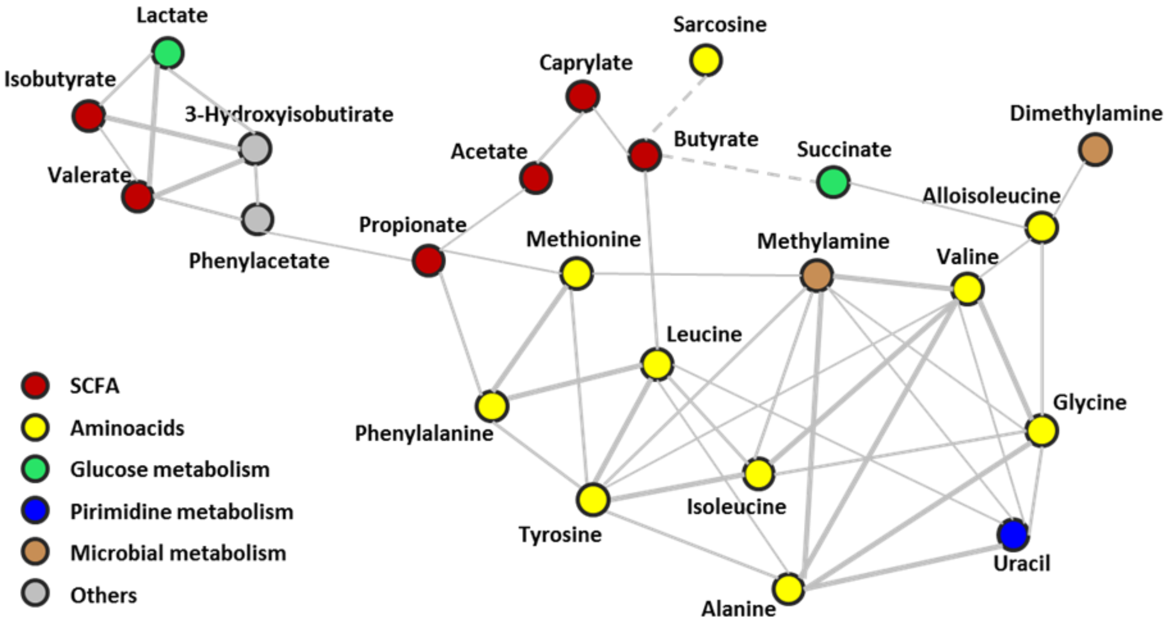

Supplemental Figure 5. Faecal metabolome analysis. Metabolite-metabolite interaction network of the faecal metabolites changes (Change at day 28 respect baseline) in subjects after 4-weeks intervention period. Only interactions with correlation values >0.800 and q< 0.05 (FDR correction) were considered. Lineal and dotted edges represent positive and negative correlations respectively and edge thickness the confidence of interaction between nodes. SCFA, short chain fatty acids.

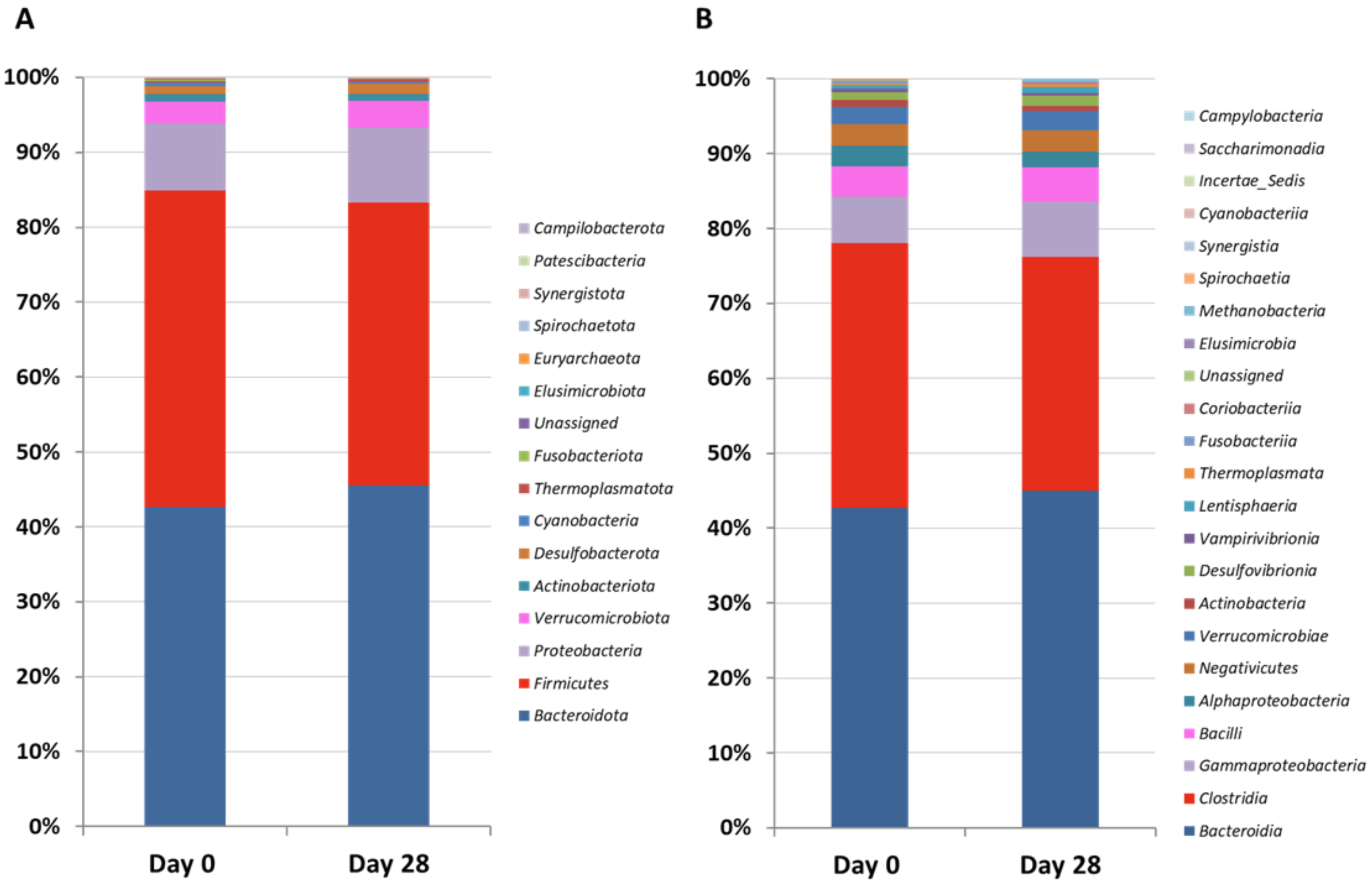

Supplemental Figure 6. Relative abundance of bacterial (A) phylas and (B) classes in fecal samples at baseline and day 28. N of each classes and phylas are indicated in supplemental table 12.

A

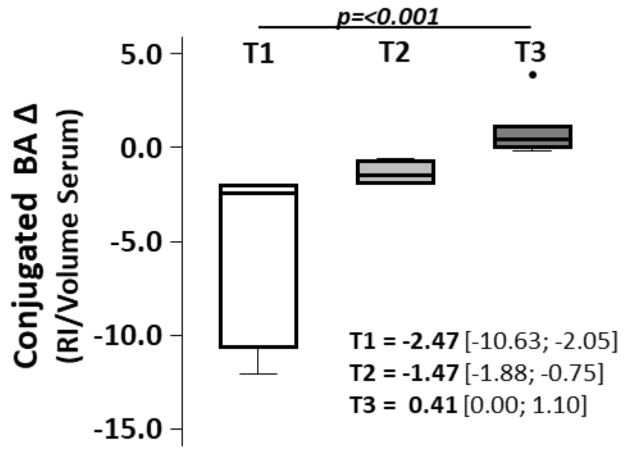

B

%	T1	T2	T3	*p-Value*
Bacteroidota				
Δ < 0 (N=7)	29	29	42	***0.015***
Δ > 0 (N=13)	39	39	22	
Firmicutes				
Δ < 0 (N=12)	33	33	34	*0.409*
Δ > 0 (N=8)	38	38	24	
Proteobacteria				
Δ < 0 (N=7)	29	57	14	***0.000***
Δ > 0 (N=13)	39	23	38	
Actinobacteria				
Δ < 0 (N=7)	14	29	57	***0.000***
Δ > 0 (N=10)	50	40	10	
Desulfobacterota				
Δ < 0 (N=4)	50	25	25	***0.003***
Δ > 0 (N=15)	27	40	33	

Supplemental Figure 7. Effect of the 4-weeks probiotic intervention on serum conjugated BA. Box plot represents tertile distribution for changes of serum conjugated BA between day 28 and baseline (T1: N=7, T2: N=7 and T3: N=6) of serum conjugated BA. Data on the table refer to the distribution of individuals in relation to the tertalies of conjugated BA. Subjects with decrease (Δ<0) and increase (Δ>0) in relative abundance of the indicated phyla after the probiotic intervention are shown separately. *p-Value were obtained by* Mann–Whitney U test (box-plot) or Chi-squared test (comparison percentage distribution). Statistical significance: $p < 0.05$. BA, bile acids; T, tertile; RI, relative abundance; Δ, change at day 28 respect baseline.
