# Supplementary material for: Lactiplantibacillus plantarum strains KABP011, KABP012, and KABP013 modulate bile acids and cholesterol metabolism in humans
Source: Cardiovasc Res. 2024 Mar 25;120(7):708–22. doi: 10.1093/cvr/cvae061 (PMC11135648; doi:10.1093/cvr/cvae061)
Supplement: cvae061_Supplementary_Data [file cvae061_supplementary_data.zip › Padro et al-STROBE_checklist_CVR-2023-0906R1.pdf]

STROBE Statement—checklist of items that should be included in reports of observational studies

	Item No	Recommendation	Page No
**Title and abstract**	1	(*a*) Indicate the study's design with a commonly used term in the title or the abstract	2 in manuscript
		(*b*) Provide in the abstract an informative and balanced summary of what was done and what was found	2 in manuscript
**Introduction**			
Background/rationale	2	Explain the scientific background and rationale for the investigation being reported	4 and 5 in manuscript
Objectives	3	State specific objectives, including any prespecified hypotheses	5 in manuscript
**Methods**			
Study design	4	Present key elements of study design early in the paper ***(registered in Clinicaltrials.gov - NCT05378230)***	6 in manuscript and 2-3 in Supp. Material & methods
Setting	5	Describe the setting, locations, and relevant dates, including periods of recruitment, exposure, follow-up, and data collection	6-7 in manuscript and 2 -3 in Supp. Material & methods
Participants	6	(*a*) *Cohort study*—Give the eligibility criteria, and the sources and methods of selection of participants. Describe methods of follow-up	6 in manuscript and 2 in Supp. Material & methods
		(*b*) *Cohort study*—For matched studies, give matching criteria and number of exposed and unexposed *Case-control study*—For matched studies, give matching criteria and the number of controls per case	Not applicable
Variables	7	Clearly define all outcomes, exposures, predictors, potential confounders, and effect modifiers. Give diagnostic criteria, if applicable (n.a)	6 to 9 in manuscript 4 to 9 in Supp. Material & methods
Data sources/ measurement	8*	For each variable of interest, give sources of data and details of methods of assessment (measurement). Describe comparability of assessment methods if there is more than one group	7 to 9 in manuscript and 4 to 9 in Supp. Material & methods
Bias	9	Describe any efforts to address potential sources of bias ***(Study limitation in Discussion Section)***	Not applicable
Study size	10	Explain how the study size was arrived at ***(It refers to a pilot exploratory study)***	Not applicable
Quantitative variables	11	Explain how quantitative variables were handled in the analyses. If applicable, describe which groupings were chosen and why	7 to 9 in manuscript and 4 to 9 in Supp. Material & methods
Statistical methods	12	(*a*) Describe all statistical methods, including those used to control for confounding	9-10 in manuscript and

			9 -10 in Supp. Material & methods
		(*b*) Describe any methods used to examine subgroups and interactions	9-10 in manuscript and 9 -10 in Supp. Material & methods
		(*c*) Explain how missing data were addressed	9 -10 in Supp. Material & methods
		(*d*) *Cohort study*—If applicable, explain how loss to follow-up was addressed ***(4-weeks interventional study -100% study population finished the study)***	3 in Supp. Material & methods
		(*e*) Describe any sensitivity analyses	Not applicable

Results

Participants	13*	(a) Report numbers of individuals at each stage of study—eg numbers potentially eligible, examined for eligibility, confirmed eligible, included in the study, completing follow-up, and analysed	11 in manuscript
		(b) Give reasons for non-participation at each stage	Not applicable
		(c) Consider use of a flow diagram	Supplemental Figure 1
Descriptive data	14*	(a) Give characteristics of study participants (eg demographic, clinical, social) and information on exposures and potential confounders	11 in manuscript
		(b) Indicate number of participants with missing data for each variable of interest	Only data under detection limit were considered as missing see supplemental tables 10, 11, 12 and 13 See also figure 5 and supp figures 5, 6 and 7
		(c) *Cohort study*—Summarise follow-up time (eg, average and total amount)	10 in manuscript
Outcome data	15*	*Cohort study*—Report numbers of outcome events or summary measures over time	10 to 16 in manuscript
		Case-control study—Report numbers in each exposure category, or summary measures of exposure	Not applicable
		Cross-sectional study—Report numbers of outcome events or summary measures	Not applicable
Main results	16	(*a*) Give unadjusted estimates and, if applicable, confounder-adjusted estimates and their precision (eg, 95% confidence interval). Make clear which confounders were adjusted for and why they were included ***(It refers to a pilot exploratory -hypothesis generating study)***	Figures 1 to 7 Pages 33-34 (legends to figures) and text in section 3 of the manuscript
		(*b*) Report category boundaries when continuous variables were categorized	Details in Supplemental Table 8 (inflammatory markers)

			Supplemental Figure 4 (LDLc) and Figure 7 (microbiota) Text description -pages 16 (inflammatory markers),14 (LDLc), 18-19 (microbiota) in manuscript
		(*c*) If relevant, consider translating estimates of relative risk into absolute risk for a meaningful time period	Not applicable
Other analyses	17	Report other analyses done—eg analyses of subgroups and interactions, and sensitivity analyses	14, 16 and 18-19 in manuscript
**Discussion**			
Key results	18	Summarise key results with reference to study objectives	20 to 24 in manuscript
Limitations	19	Discuss limitations of the study, taking into account sources of potential bias or imprecision. Discuss both direction and magnitude of any potential bias	24 in manuscript
Interpretation	20	Give a cautious overall interpretation of results considering objectives, limitations, multiplicity of analyses, results from similar studies, and other relevant evidence	24 and 25 in manuscript
Generalisability	21	Discuss the generalisability (external validity) of the study results	20 to 24 in manuscript
**Other information**			
Funding	22	Give the source of funding and the role of the funders for the present study and, if applicable, for the original study on which the present article is based	26 in manuscript

*Give information separately for cases and controls in case-control studies and, if applicable, for exposed and unexposed groups in cohort and cross-sectional studies.

Note: An Explanation and Elaboration article discusses each checklist item and gives methodological background and published examples of transparent reporting. The STROBE checklist is best used in conjunction with this article (freely available on the Web sites of PLoS Medicine at http://www.plosmedicine.org/, Annals of Internal Medicine at http://www.annals.org/, and Epidemiology at http://www.epidem.com/). Information on the STROBE Initiative is available at www.strobe-statement.org.
